# MgrB Inactivation Confers Trimethoprim Resistance in *Escherichia coli*

**DOI:** 10.3389/fmicb.2021.682205

**Published:** 2021-07-28

**Authors:** Hongmei Shi, Ting Li, Jintian Xu, Jifang Yu, Shanshan Yang, Xian-En Zhang, Shengce Tao, Jing Gu, Jiao-Yu Deng

**Affiliations:** ^1^CAS Key Laboratory of Special Pathogens and Biosafety, Wuhan Institute of Virology, Chinese Academy of Sciences, Wuhan, China; ^2^University of Chinese Academy of Sciences, Beijing, China; ^3^National Laboratory of Biomacromolecules, Institute of Biophysics, Chinese Academy of Sciences, Beijing, China; ^4^Shanghai Center for Systems Biomedicine, Key Laboratory of Systems Biomedicine (Ministry of Education), Shanghai Jiao Tong University, Shanghai, China; ^5^Guangdong Province Key Laboratory of TB Systems Biology and Translational Medicine, Foshan, China

**Keywords:** *Escherichia coli*, MgrB, PhoP/Q, *folA*, gene expression regulation, trimethoprim resistance

## Abstract

After several decades of use, trimethoprim (TMP) remains one of the key access antimicrobial drugs listed by the World Health Organization. To circumvent the problem of trimethoprim resistance worldwide, a better understanding of drug-resistance mechanisms is required. In this study, we screened the single-gene knockout library of *Escherichia coli*, and identified *mgrB* and other several genes involved in trimethoprim resistance. Subsequent comparative transcriptional analysis between Δ*mgrB* and the wild-type strain showed that expression levels of *phoP*, *phoQ*, and *folA* were significantly upregulated in Δ*mgrB*. Further deleting *phoP* or *phoQ* could partially restore trimethoprim sensitivity to Δ*mgrB*, and co-overexpression of *phoP/Q* caused TMP resistance, suggesting the involvement of PhoP/Q in trimethoprim resistance. Correspondingly, MgrB and PhoP were shown to be able to modulated *folA* expression *in vivo*. After that, efforts were made to test if PhoP could directly modulate the expression of *folA*. Though phosphorylated PhoP could bind to the promotor region of *folA in vitro*, the former only provided a weak protection on the latter as shown by the DNA footprinting assay. In addition, deleting the deduced PhoP box in Δ*mgrB* could only slightly reverse the TMP resistance phenotype, suggesting that it is less likely for PhoP to directly modulate the transcription of *folA*. Taken together, our data suggested that, in *E. coli*, MgrB affects susceptibility to trimethoprim by modulating the expression of *folA* with the involvement of PhoP/Q. This work broadens our understanding of the regulation of folate metabolism and the mechanisms of TMP resistance in bacteria.

## Introduction

Folic acid is essential for the survival of various living organisms. Unlike bacteria, mammals can take folic acid from food without creating folic acid from *de novo* synthesis. Therefore, the key enzyme in bacterial *de novo* synthesis of folic acid is an ideal target for antibacterial drug design (Bermingham and Derrick, 2002). To date, thousands of folate antagonists have been designed for bacterial dihydropteroate synthase (DHPS) and dihydrofolate reductase (DHFR) (Estrada et al., 2016), of which sulfamethoxazole (SMX) and trimethoprim (TMP) are most widely used. TMP can specifically inhibit the activity of DHFR and inhibit the growth of bacteria by blocking the synthesis of folate (Hitchings, 1973). TMP was first used clinically in 1962 in the treatment of *Proteus septicemia* (Noall et al., 1962). Since 1967, Co-trimoxazole, a fixed-dose combination of trimethoprim (TMP) and SMX, has been developed and used as a broad-spectrum antibacterial drug (Bushby and Barnett, 1967; Csonka and Knight, 1967; Akinkugbe et al., 1968; Bushby and Hitchings, 1968; Reeves, 1971). Initially, co-trimoxazole was used to treat urinary tract infections and lower respiratory tract infections (Hughes, 1969; Reeves et al., 1969), but later on, it was found to have good prophylactic activity against *Pneumocystis jiroveci* pneumonia (Hughes et al., 1974). The use of co-trimoxazole greatly increased with the emergence of the HIV epidemic in the 1980s since it provided good protection against various opportunistic infections during the pre-antiretroviral therapy (ART) era (DeHovitz et al., 1986; Youle et al., 1990; Canessa et al., 1992; Carr et al., 1992; Plourde et al., 1992; Schneider et al., 1992). In recent years, researchers have found that co-trimoxazole is able to prevent the development of tuberculosis in HIV-positive patients, especially in those who have not received prior ART (Hasse et al., 2014).

However, the widespread use of co-trimoxazole has resulted in increasing resistance, just like many other antimicrobial agents (Huovinen et al., 1995). So far, known mechanisms of TMP resistance include: (1) plasmid-mediated drug-insusceptible variants of chromosomal dihydrofolate reductase (DHFR) (Fleming et al., 1972; Amyes and Smith, 1974; Skold and Widh, 1974; Then, 1993), which was considered as the target protein of the TMP, encoded by *folA* in *Escherichia coli*; (2) chromosomal location of transposon Tn7 (Lichtenstein and Brenner, 1981); (3) Mutation of the thymidylate synthase encoding gene *thyA* (Chatterjee et al., 2008); (4) overproduction of DHFR, or alteration of the enzyme, or a combination of both (Sirotnak and McCuen, 1973; Sheldon and Brenner, 1976; Smith and Calvo, 1982; Palmer and Kishony, 2014). Even so, at present, co-trimoxazole is still one of the first-line drugs for the treatment of urinary tract infections, skin and soft tissue infections, and community-acquired methicillin resistant *Staphylococcus aureus* infections (Gupta et al., 2011). To combat the problem, it is important to understand resistance-mechanism and accordingly develop alternative strategies.

The PhoP/Q pathway is a well-known two-component signaling system found in *Enterobacteriaceae*, such as *E. coli*, *Salmonella typhimurium*, and related bacteria. PhoQ, a transmembrane histidine kinase, is stimulated by exposure to low extracellular Mg^2+^, certain cationic antimicrobial peptides, and low pH (Groisman, 2001; Bader et al., 2005; Cho et al., 2006). Once activated, PhoQ autophosphorylates and transfers the phosphoryl group to PhoP, which then induces the expression of its target genes (Igo et al., 1989). It has been proposed that phosphorylated PhoP binds to a hexanucleotide direct repeat [(T/G)GTTTA] separated by five nucleotides, the so-called “PhoP box” (Kato et al., 1999). The PhoP box was located at various distances from the −10 region recognized by RNA polymerase in the promoters of PhoP target genes (Kato et al., 1999; Yamamoto et al., 2002; Minagawa et al., 2003; Zwir et al., 2005, 2012; Li et al., 2008; Zhao et al., 2008; Harari et al., 2010). One of the targets of PhoP is MgrB, a broadly conserved small transmembrane protein, which inhibits the kinase activity of PhoQ via a negative feedback loop (Lippa and Goulian, 2009). PhoQ is bifunctional: when its kinase activity is inhibited, it induces the dephosphorylation of PhoP (Yeo et al., 2012). Thus, MgrB drives the inactivation of PhoP (Lippa and Goulian, 2009; Salazar et al., 2016). Previous studies have demonstrated that the PhoP/Q pathway functions as a critical part of the survival stress response under disparate environmental conditions (Eguchi et al., 2011); this pathway is also involved in drug resistance and virulence in many pathogenic bacteria (Gunn and Miller, 1996; Gunn et al., 1998; Mitrophanov et al., 2008; Cheng et al., 2010; Cannatelli et al., 2013, 2014; Hicks et al., 2015; Aires et al., 2016). Recently, it has been shown that MgrB is also involved in the resistiance of *Klebsiella pneumoniae* to polymyxins (polymyxin B and colistin, also known as polymyxin E) (Cannatelli et al., 2013; Aires et al., 2016).

In this study, many genes involved in TMP resistance, including *mgrB*, were identified by screening the *E. coli* single-gene knockout library (Baba et al., 2006). And the mechanism of TMP resistance induced by deletion of *mgrB* was investigated by subsequent microbiological and biochemical experiments.

## Results

### Screening of Genes Associated With TMP Susceptibility

To identify genes in the *E. coli* genome potentially involved in resistance or susceptibility to TMP, we screened 3985 single-gene knockout mutants of *E. coli* K-12 BW25113 (the Keio collection). Deletion of 31 of these genes led to changes in TMP susceptibility (Supplementary Table 1). To verify these results, all in-frame deletion mutants were reconstructed in *E. coli* K-12 W3110, along with the corresponding overexpression and complemented gene strains. We tested the susceptibility of these strains to TMP. We found that the deletion of five genes (*rfaH*, *acrB*, *acrA*, *phoP*, and *phoQ*) increased sensitivity to TMP, while the deletion of six other genes (*mgrB*, *ymjC*, *rcsC*, *tehB*, *cysB*, and *torR*) decreased sensitivity to TMP (Table 1). Of the deletion mutants tested, two (*acrA* and *acrB*) encode drug efflux proteins (Ma et al., 1995), which might affect TMP susceptibility. However, all other tested genes encode proteins which are not directly related to bacterial drug resistance. For example, *ymjC* encodes an putative oxidoreductase; *rcsC* encodes a histidine kinase of the RCS regulatory cascade; *tehB* encodes a tellurite methyltransferase; *rfaH* encodes a transcription antitermination protein; *cysB* encodes a transcriptional regulator involved in sulfur utilization; and *torR* encodes the response regulator of the TorT-TorS-TorR signal transduction system.

**TABLE 1:** Genes associated with TMP susceptibility in *E. coli* K-12 W3110 strains.

**Gene name**	**TMP MICs (μg ml^–1^) of wild-type strains**	**TMP MICs (μg ml^–1^) of gene deletion mutants**	**TMP MICs (μg ml^–1^) of complemented strains**	**TMP MICs (μg ml^–1^) of overexpressed strains**
*mgrB*	0.16	1.28	0.16	0.16
*ymjC*	0.16	0.32	0.32	0.16
*rcsC*	0.16	0.32	0.16	0.16
*tehB*	0.16	0.32	0.16	0.16
*rfaH*	0.16	0.08	0.08	0.16
*pgpA*	0.16	0.16	0.64	0.64
*cysB*	0.16	0.64	0.16	0.16
*torR*	0.16	0.64	0.64	0.16
*phoP*	0.16	0.08	0.16	0.16
*acrB*	0.16	0.04	0.08	0.16
*yhfK*	0.16	0.16	0.08	0.08
*acrA*	0.16	0.02	0.04	0.16
*phoQ*	0.16	0.08	0.16	0.16

*Cultures were incubated overnight at 37°C on LB with 10 μM IPTG.*

The Δ*mgrB* mutant was shown to be moderately more resistant to TMP (an eight-fold increase in TMP MIC compared with the wild-type strain), while the Δ*phoP* and Δ*phoQ* mutants were slightly more sensitive to TMP (a two-fold decrease in TMP MIC compared with the wild-type strain).

### Deletion of *phoP* or *phoQ* Restores TMP Sensitivity to Δ*mgrB*

It is known that *mgrB* encodes MgrB, which feeds back to inhibit the kinase activity of PhoQ, so the MgrB-dependent negative feedback loop plays a crucial role in *E. coli* PhoQ-PhoP signaling (Lippa and Goulian, 2009; Salazar et al., 2016). We thus hypothesized that the TMP-resistant phenotype resulting from *mgrB* deletion might be mediated by the PhoP/Q pathway. To prove this hypothesis, we constructed two double-knockout mutants (*E. coli* W3110 Δ*mgrB* Δ*phoQ* and *E. coli* W3110 Δ*mgrB* Δ*phoP*) and tested their susceptibilities to TMP. The results showed that the TMP MICs of both double knockout mutants were identical to that of the wild-type strain, two times that of Δ*phoP* or Δ*phoQ*, demonstrating that disruption of the PhoP/Q pathway could partially reversed the effects of *mgrB* deletion (Table 2).

**TABLE 2:** MICs (μg ml^–1^) to TMP in different strains of *E. coli* K-12 W3110.

**Strain**	**Description**	**MIC**
**phoP/Q pathway-related strains**		
W3110 pCA24N	W3110 transformed with pCA24N	0.16
W3110 Δ*mgrB* Δ*phoP* pCA24N	W3110 Δ*mgrB* Δ*phoP* transformed with pCA24N	0.16
W3110 Δ*mgrB* Δ*phoQ* pCA24N	W3110 Δ*mgrB* Δ*phoQ* transformed with pCA24N	0.16
W3110 Δ*mgrB* pCA24N	W3110 Δ*mgrB* transformed with pCA24N	1.28
W3110 Δ*phoP* pCA24N	W3110 Δ*phoP* transformed with pCA24N	0.08
W3110 Δ*phoQ* pCA24N	W3110 Δ*phoQ* transformed with pCA24N	0.08
W3110 pCA24N:*phoPQ*	W3110 transformed with pCA24N:*phoPQ*	0.64
W3110 pCA24N:*phoQ*	W3110 transformed with pCA24N:*phoQ*	0.16
W3110 pCA24N:*phoP*	W3110 transformed with pCA24N:*phoP*	0.08
W3110 pCA24N:*folA*	W3110 transformed with pCA24N:*folA*	10.24
*pmrK* related strains		
W3110 pCA24N	W3110 transformed with pCA24N	0.16
W3110 Δ*pmrK* pCA24N	W3110 Δ*pmrK* transformed with pCA24N	0.16
W3110 pCA24N:*pmrK*	W3110 transformed with pCA24N:*pmrK*	0.16
W3110 Δ*pmrK* pCA24N:*pmrK*	W3110 Δ*pmrK* transformed with pCA24N:*pmrK*	0.16
*tolC* related strains		
W3110 pCA24N	W3110 transformed with pCA24N	0.16
W3110 pCA24N:*tolC*	W3110 transformed with pCA24N:*tolC*	0.16

*Cultures were incubated overnight at 37°C on LB with 10 μM IPTG.*

### Deletion of *pmrK* Does Not Affect Susceptibility of *E. coli* to TMP

Previous studies have shown that the deletion of *mgrB* led to the upregulation of the PhoQ/PhoP pathway and the activation of *pmrK* (part of *pmrHFIJKLM* operon), eventually causing resistance to polymyxins via modifications of the lipopolysaccharide target in *K. pneumoniae* (Helander et al., 1996; Cannatelli et al., 2013). To investigate whether *pmrK* was also involved in the TMP resistance of *E. coli* Δ*mgrB*, we both knocked out and overexpressed *pmrK* in *E. coli* W3110. Our results showed that *pmrK* did not affect TMP susceptibility in *E. coli* (Table 2). In addition, the Δ*mgrB* mutant was not more resistant to polymyxin B or colistin than was the wild-type strain (Table 3).

**TABLE 3:** MICs (μg ml^–1^) to polymyxins in *mgrB-*related and *phoP-*related strains of *E. coli* K-12 W3110.

**Strain**	**Description**	**MIC to polymyxin B**	**MIC to colistin**
W3110 pCA24N	W3110 transformed with pCA24N	0.64	0.16
W3110 Δ*mgrB* pCA24N	W3110 Δ*mgrB* transformed with pCA24N	0.64	0.16
W3110 pCA24N:*mgrB*	W3110 transformed with pCA24N:*mgrB*	0.32	0.16
W3110 Δ*mgrB* pCA24N:*mgrB*	W3110 Δ*mgrB* transformed with pCA24N:*mgrB*	0.64	0.16
W3110 Δ*phoP* pCA24N	W3110 Δ*phoP* transformed with pCA24N	0.16	0.04
W3110 pCA24N:*phoP*	W3110 transformed with pCA24N:*phoP*	0.64	0.16
W3110 Δ*phoP* pCA24N:*phoP*	W3110 Δ*phoP* transformed with pCA24N:*phoP*	0.64	0.16

*Cultures were incubated overnight at 37°C on LB with 10 μM IPTG.*

### Overexpression of *tolC* Does Not Affect Susceptibility of *E. coli* to TMP

**FIGURE 1:**
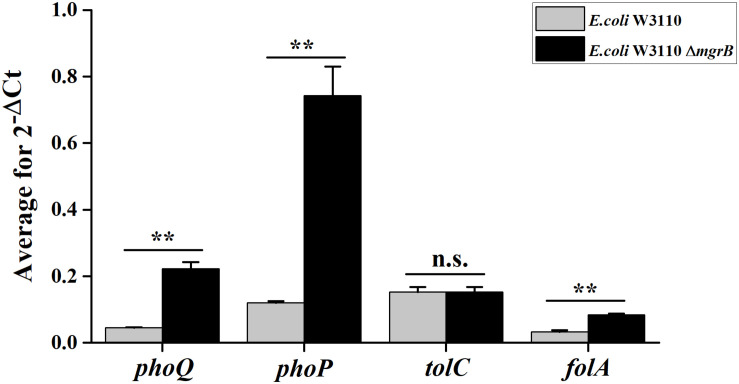
Average transcription levels of the genes *phoP*, *phoQ*, *folA*, and *tolC* in *E. coli* W3110 (wild type) and *E. coli* W3110 Δ*mgrB*, as measured with qRT-PCR. Expression levels of *gapA* were normalized as an endogenous control. Data represent mean ± SD from three independent experiments. *P* values were calculated using *t* tests (*P < 0.05; **P < 0.01; ***P < 0.001).

TolC, an outer membrane protein, is part of the AcrAB-TolC drug efflux system (Tikhonova and Zgurskaya, 2004). This system plays an important role in both intrinsic and acquired resistance to a wide variety of currently available antimicrobial agents (Du et al., 2014). Previous studies have identified a putative PhoP binding site (PhoP box) in the *tolC* promoter region (Eguchi et al., 2003). As our results indicated that PhoP/Q was involved in the TMP resistance of the Δ*mgrB* mutant, we speculated that the PhoP/Q pathway might affect TMP sensitivity by modulating the expression of *tolC*. To test this theory, we overexpressed *tolC* in *E. coli* W3110. We found that the overexpression of *tolC* did not affect TMP susceptibility (Table 2). In addition, our qRT-PCR results suggested that the deletion of *mgrB* did not affect *tolC* expression (Figure 1).

### Transcriptome Analysis of the Δ*mgrB* Mutant

To further understand how *mgrB* deletion led to TMP resistance in *E. coli*, we compared the global transcription profiles of the *E. coli* K12 W3110 Δ*mgrB* mutant and the wild-type *E. coli* strain using RNA-seq (PRJNA523715). We identified 518 significantly upregulated genes and 433 significantly downregulated genes (951 in total; Supplementary Table 2). As expected, the deletion of *mgrB* was followed by a significant upregulation of *phoQ* and *phoP*. Interestingly, *folA* also was upregulated. The *folA* gene encodes the DHFR of *E. coli*, the target of TMP (Table 4). We verified these results with qRT-PCR (Figure 1).

**TABLE 4:** Genes differentially regulated in *E. coli* K-12 W3110 Δ*mgrB* compared with wild-type *E. coli* K-12 W3110 by comparative transcriptional analyses.

**Gene name**	**Description**	**Fold changes**	**Tendency**
*folA*	Dihydrofolate reductase	3.57	up
*folM*	Dihydrofolate reductase isozyme	2.18	up
*phoP*	DNA-binding response regulator in two-component regulatory system with PhoQ	7.61	up
*phoQ*	Sensory histidine kinase in two-component regulatory system with PhoP	6.74	up
*pmrD*	Polymyxin resistance protein B	0.31	down

### Co-overexpression of *phoP* and *phoQ* Causes TMP Resistance

To further test the involvement of PhoP/Q in the TMP resistant phenotype of Δ*mgrB*, we constructed three strains of *E. coli* W3110, overexpressing *phoP*, *phoQ*, or *phoPQ*, and tested their sensitivity to TMP. Our results showed that while overexpressing *phoP* or *phoQ* did not affect TMP sensitivity, co-overexpression of *phoP* and *phoQ* caused TMP resistance (Table 2).

### PhoP Recognizes the *folA* Promoter *in vitro*

**FIGURE 2:**
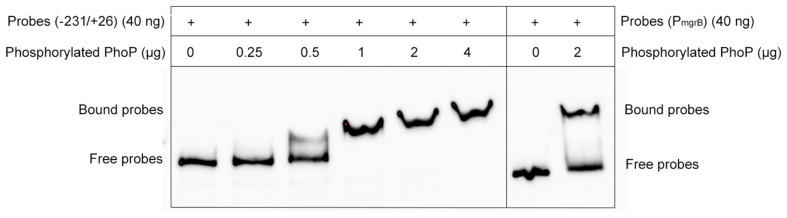
Electrophoretic mobility shift assays (EMSAs) of PhoP to the *folA* promoter region. A varying concentration of the PhoP (0, 0.25, 0.5, 1, 2, and 4 μg) was incubated with PCR-amplified biotin-labeled probes of the promoter regions of *folA*, and the binding of PhoP (2 μg) to the promoter regions of *mgrB* was used as the positive control, showing that PhoP recognized and bound to the promoter of *folA*. The experiment was repeated at least three times and a representative result was presented.

As the deletion of *mgrB* was followed by the increased expression of *folA*, *phoP*, and *phoQ*, we hypothesized that MgrB might modulate the expression of *folA* via the PhoP/Q pathway. To verify this hypothesis, we used electrophoretic mobility shift assay (EMSA) assay to test whether PhoP recognized and bound the promoter regions of *folA* directly, by using the promoter region of *mgrB*, a known PhoP substrate (Kato et al., 1999), as the positive control. Phosphorylated PhoP with a poly-histidine tag was prepared, and the biotin-labeled DNA probe containing the promoter region of *folA* (−231/+26) were amplified by PCR using 3′end biotin-labeled primers. After combined incubation and subsequent electrophoresis, it was clearly that PhoP hindered the mobility of the probe, and the hindering effect increased correlating to the quantities of PhoP, suggesting that PhoP specifically recognize and bind the promoter region of *folA* (Figure 2).

### MgrB and PhoP Regulate *folA* Gene Expression *in vivo*

**FIGURE 3:**
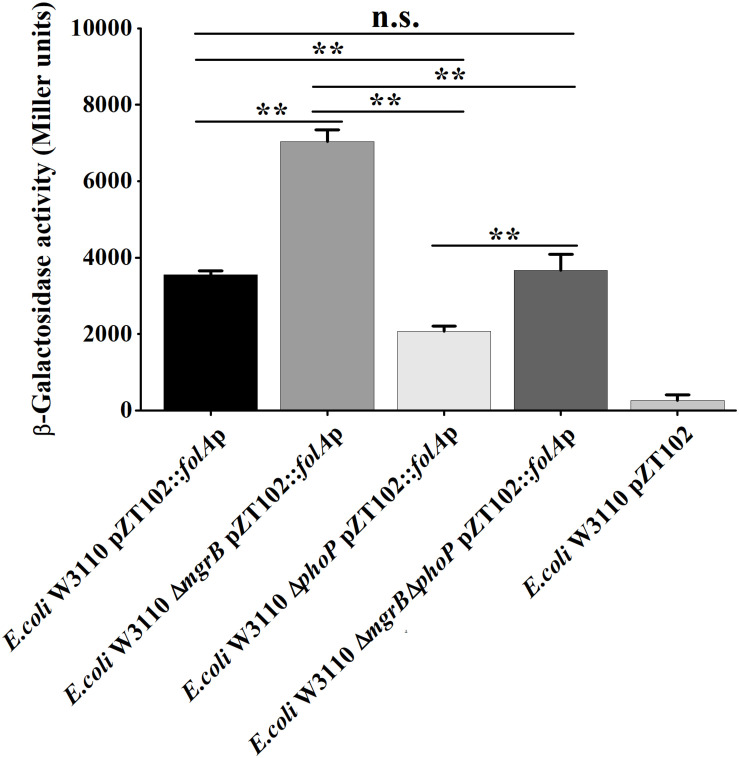
A comparison of promoter activity of *folA* in various strains of *E. coli* using *lacZ*-fusion analysis. β-Galactosidase activities were measured from exponential phase aerobic cultures in LB of several *E. coli* W3110 strains: wild-type (WT), null mutants (Δ*phoP* and Δ*mgrB*), and a double mutant (Δ*mgrB* Δ*phoP*), all of them carrying a reporter plasmid pZT102:*folA*p-257 containing the promoter region of *folA* (–231/+26). CK is empty pZT102 plasmid control in *E. coli* W3110. Means and standard deviations are represented. Data represent mean ± SD from three independent experiments. *P* values were calculated using *t* tests (*P < 0.05; **P < 0.01; ***P < 0.001).

To confirm that MgrB and PhoP regulated the expression of *folA*, the β-galactosidase activities of strains (the wild type, the Δ*phoP* mutant, the Δ*mgrB* mutant, and the Δ*mgrB* Δ*phoP* mutant) harboring a reporter plasmid pZT102 (Li et al., 2014) (Supplementary Figure 1) carrying a *lacZ* gene fusion with the *folA* promoter region (−231/+26) were determined during the exponential growth phase. Our results showed that deletion of *mgrB* caused a ∼2-fold increase in the β-galactosidase activity, and deletion of *phoP* led to about 40% decrease of the β-galactosidase activity (Figure 3). Further deletion of *phoP* gene in the Δ*mgrB* mutant decreased the β-galactosidase activity back to the level of the wild type strain, but not to that of the Δ*phoP* mutant.

### Determination of the PhoP-Binding Sites in the Promoter of *folA*

**FIGURE 4:**
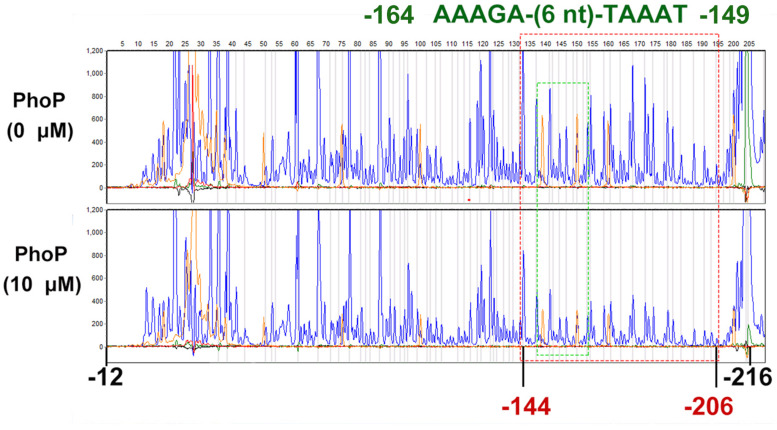
DNase I footprinting assays for mapping the binding sequence recognized by PhoP. FAM-labeled primer-based DNase footprinting experiments. Protection of the *folA* promoter DNA against DNase digestion by phosphorylated PhoP (10 μM) was evaluated. The sequences of the protected regions on the non-coding strand are framed in the figures. The fluorescence signal faded out obviously from position −144 to the end of the strand, but not completely disappeared (Dotted red box). Dotted green box contains a tandem repeat sequence AAAGA-(6 nt)-TAAAT (−164 to −149). The experiment was repeated at least three times and a representative result was presented.

To determine the PhoP-binding site in the *folA* promoter, DNase I footprinting assay was employed. The non-coding strand of the *folA* gene (−216 to −12) was labeled with FAMs and utilized as substrate for the assay. The mixture of phosphorylated PhoP and the substrate was co-incubated with DNase I, and then protected regions were analyzed. The results showed that the fluorescence signal faded out obviously from position −144 to the end of the strand, but not completely disappeared (Figure 4).

**FIGURE 5:**
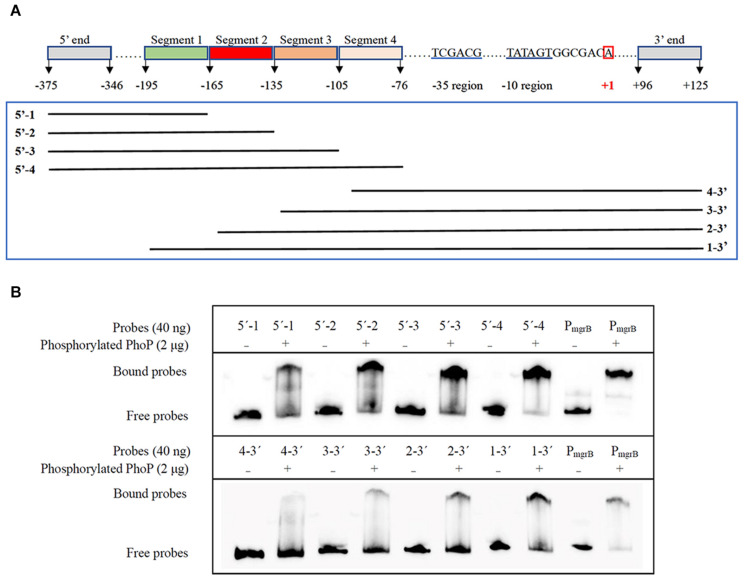
Determination of the PhoP-binding fragment in the promoter of *folA* using EMSA assay. **(A)** Nucleotide sequence of the *folA* promoter regulatory region. The numbering of the sequence is relative to the start site of transcription indicated with red box. The –35 and–10 regions of the *folA* promoter are underlined in blue. Segment 1 (–195/–166), segment 2 (–165/–136), segment 3 (–135/–106), and segment 4 (–105/–76) are indicated with highlights or underlines. The two sets of probes of gradually shortened *folA* promoter fragments are framed in green. **(B)** Binding of PhoP to the probes showed in **(A)**, and the binding of PhoP to the promoter regions of *mgrB* was used as the positive control. The experiment was repeated at least three times and a representative result was presented.

Further EMSA assays were performed to identify the precise PhoP binding sites. Another two sets of gradually truncated *folA* promoter fragments in the direct and reverse orientations were prepared (Figure 5A). The results showed that more phosphorylated PhoP bound to the fragments containing segment 2 (−165 to −136) than those without segment 2, suggesting that segment 2 plays an important role on PhoP binding to the promoter region of *folA* (Figure 5B).

To further determine the PhoP binding sites in segment 2, sequences alignment of the known “reverse PhoP boxes” (Supplementary Figure 2) was performed (Zwir et al., 2005, 2012). The results of the footprinting experiment also showed that fluorescence signals attenuated more significantly from position −165 to −155 (Supplementary Table 3). Thus, a tandem repeat sequence AAAGA-(6 nt)-TAAAT (−164 to −149) was deduced to be a putative “reverse PhoP box” in the promotor region of *folA*.

**FIGURE 6:**
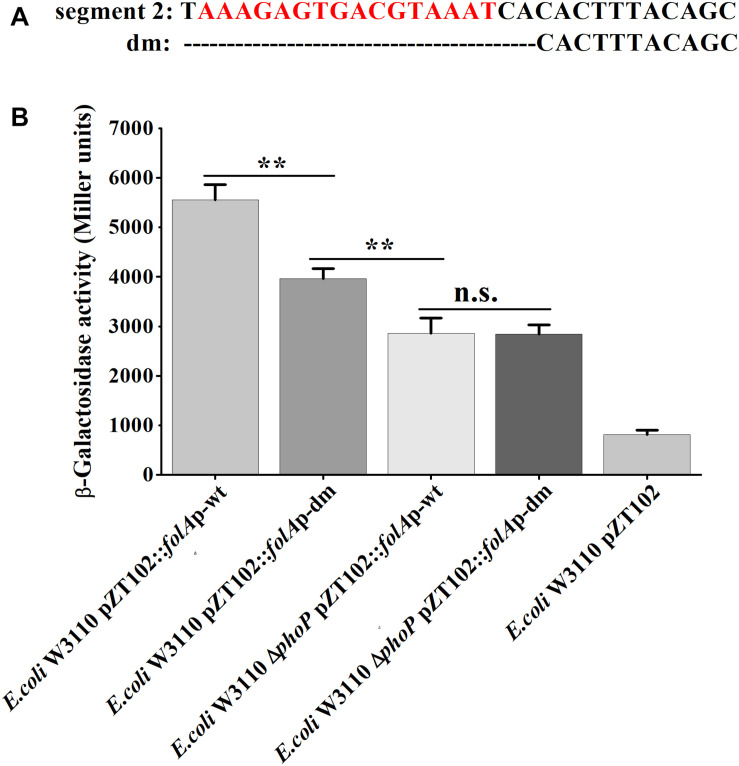
Determination of the PhoP-binding sites in the promoter of *folA*. **(A)** The deletion mutant was designed in segment 2 of the promotor region of *folA*, designated as dm. The predicted putative “reverse PhoP box” is indicated with red letters. **(B)** β-Galactosidase activities were measured from exponential phase aerated cultures in LB of the wild-type *E. coli* W3110 strain and Δ*phoP* mutant strain. They either carry the pZT102 reporter plasmids containing the promotor region of *folA* (–165/+26) or its mutants (dm). Data represent mean ± SD from three independent experiments. *P* values were calculated using *t* tests (*P < 0.05; **P < 0.01; ***P < 0.001).

Furthermore, mutational analysis was carried out to test the above deduced “reverse PhoP box.” A deletion mutant (−166 to −147 deleted, abbreviated to “dm”) of the *folA* promotor were designed and ligated to pZT102 (Figure 6). The results of subsequent β-galactosidase reporter assay showed that, deletion of the predicted “reverse PhoP box” led to decreased transcriptional activity of the *folA* promotor (Figure 6). The transcriptional activity of the *folA* promotor with deletion of the predicted “reverse PhoP box” tested in W3110 was shown to be slightly but significantly higher to that of the intact *folA* promotor tested in the *phoP* deletion mutant. On the other hand, deletion of the predicted “reverse PhoP box” led to an about 30% decrease of the transcriptional activity of the *folA* promotor when tested in W3110, but no difference could be observed when tested in the *phoP* deletion mutant.

Meanwhile, we also replaced the promoter region of *folA* in the chromosome of *E. coli* W3110 Δ*mgrB* with the promoter fragments having dm mutation, or the wild type promoter fragment, and measured TMP susceptibilities of the three recombinant strains. Our results showed that deletion of the predicted “reverse PhoP box” in the *folA* promotor led to slightly increased sensitivity to TMP (Table 5).

**TABLE 5:** MICs (μg ml^–1^) to TMP in different strains of *E. coli* K-12 W3110.

**Strain**	**Description**	**MIC**
W3110	*E. coli* W3110	0.16
W3110 Δ*mgrB*	*E. coli* W3110 Δ*mgrB*	1.28
W3110 Δ*mgrB* ΔP*_*phoP*_*:P*_*phoP*_*	*E. coli* W3110 Δ*mgrB* replacing the promoter region of *folA* in the chromosome with the wild type promoter of *folA*	1.28
W3110 Δ*mgrB* ΔP*_*phoP*_*: dm-P*_*phoP*_*	*E. coli* W3110 Δ*mgrB* replacing the promoter region of *folA* in the chromosome with the deletion mutated promoter of *folA*	0.64

*Cultures were incubated overnight at 37°C on LB.*

## Discussion

Antifolates have been extensively used as anti-infective drugs for decades, but the mechanisms of antifolates resistance are not yet fully understood. In a recent screening of a library of transposon mutants, about 50 chromosomal loci were shown to be involved in the intrinsic antifolate resistance of *Mycobacterium smegmatis* (Guzzo et al., 2016). Here, we screened the *E. coli* Keio (single-gene, knockout mutants) library, and several new determinants of TMP resistance were also identified, including MgrB, a negative regulator of the PhoP/Q pathway.

As expected, deleting *mgrB* caused up-regulation of the PhoP/Q regulon, since MgrB is known to inhibit the enzymatic activity of PhoQ. Further deleting *phoP* or *phoQ* could partially reverse the TMP resistance phenotype of Δ*mgrB* mutant, and co-overexpression of phoP/Q caused TMP resistance, suggesting the involvement of the PhoP/Q system in mediating the TMP resistance phenotype of Δ*mgrB* mutant. However, the downstream effector needs to be identified. From the comparative transcriptome analysis, we found that deleting *mgrB* caused increased expression of *folA*, which was verified by subsequent RT-PCR and β-gal assays. Meanwhile, deleting *phoP* led to decreased expression of *folA*. The *folA* gene encodes the DHFR in *E. coli*, which is the target of TMP. Thus, over-expression of *folA* is the direct cause of TMP resistance in Δ*mgrB*. These data indicates the connection of the PhoP/Q system and folate metabolism in *E. coli*. To further probe the potential clinical relevance of this work, the *mgrB* gene was also deleted in O157, but that did not affect the susceptibility to TMP (Supplementary Table 4.). Subsequent RT-PCR analysis showed that, deleting *mgrB* did not affect the expression levels of *phoP/Q* (Supplementary Figure 3), suggesting the defect of feedback inhibition of PhoP/Q by MgrB in O157.

Though it is not clear why the PhoP/Q system is connected with folate metabolism in *E. coli*, clues could be found in previous works. Lippa and Goulian (2012) found that, except for low extracellular Mg^2+^, cationic antimicrobial peptides, and low pH, perturbation of the oxidizing environment of the periplasm can also stimulate the PhoP/Q system in *E. coli*, which is mediated by MgrB. Later, Cardenal-Munoz and Ramos-Morales (2013) showed that in *Salmonella enterica*, cellular redox status contributes to the regulation of genes regulated by the PhoQ/PhoP system. On the other hand, Waller et al. (2010) discovered that, tetrahydrofolate plays a role on the metabolism of iron-sulfur clusters in all domains of life and hence connects with oxidative stress response. So, the PhoP/Q system may possibly be linked with folate metabolism by sensing the cellular redox status of *E. coli*.

As mentioned above, though PhoP/Q was shown to be involved in modulating the transcription of *folA* by MgrB, further deleting *phoP* could only partially reverse the TMP resistance phenotype of Δ*mgrB*. This suggests that though MgrB can modulate the transcripton of *folA* through PhoP/Q, it can also do that through another way, which merits further investigation. Previously, we found that deleting *mgrB* caused acid resistance in *E. coli*, and further deleting of *phoP* could partially reverse the phenotype of Δ*mgrB* (Xu et al., 2019). Recently, we found that, MgrB can also affect acid stress response of *E. coli* through TyrR (will be published in another manuscript), a known transcriptional factor of *folA* (Yang et al., 2007). So it is possible that MgrB might also affect the transcription of *folA* through TyrR, which merits further investigation.

Next, we tried to see if PhoP could directly modulate the transcription of *folA* in *E. coli*. Though the data of EMSA experiments showed that phosphorylated PhoP could bind to the promotor region of *folA in vitro*, the former could only provide weak protection to the latter in the subsequent DNA footprinting experiments. Since it was not possible to locate the precise PhoP binding sites from the data of DNA footprinting analysis, we chose to combine our data with the data of sequences alignments of published “PhoP box” (Zwir et al., 2005, 2012; Harari et al., 2010), and then deduced a putative “reverse PhoP box” (AAAGA-(6 nt)-TAAAT, −164 to −149). However, deleting the deduced “reverse PhoP box” in the promotor region of *folA* could only slightly reverse the TMP resistance phenotype. It is difficult to determine whether this is really caused by disrupting the binding of PhoP to the promotor region of *folA*. From these data, we think it is less likely that PhoP can directly modulate the transcription of *folA* in *E. coli* though the possibility cannot be completely ruled out. More works are required to elucidate how PhoP modulates the transcription of *folA*.

Though *folA* has been shown to be a member of the TyrR regulon, the role of TyrR in folate metabolism remains unclear. Except for TyrR, no other transcriptional regulator of *folA* has ever been identified so far. Here, we show that PhoP can modulate the transcription of *folA*, though the fine mechanism remains to be uncovered.

Taken together, in this manuscript, we show that MgrB can modulate the transcription of *folA* in *E. coli*, thus affect the susceptibility to TMP, an important broad-spectrum antimicrobial drugs extensively used in clinic. Though MgrB can modulate the expression of *folA* through PhoP/Q, it may also achieve this through other pathway. From the data obtained, it is more likely that PhoP modulates the transcription of *folA* from an indirect way, which merits further investigation. Previously, MgrB has been shown to be involved in polymyxin resistance in *K. pneumoniae*. Here we show that MgrB is an important determinant of TMP resistance in *E. coli*. These results broaden our understanding of the mechanisms of the regulation of folate metabolism, as well as the mechanisms of TMP resistance in bacteria. Because *E. coli* is a widespread pathogenic bacterium, it is vital to further explore the role of *mgrB* mutations on TMP resistance in clinical isolates of pathogenic *E. coli* strains.

## Materials and Methods

### Bacterial Strains, Plasmids, and Culture Conditions

All plasmids and strains used in this study are listed in Supplementary Table 5. Strains of *E. coli* were cultured in Luria-Bertani (LB) medium (Difco) at 37°C. Plasmids pKD4, pKD46, and pCP20 were used for the construction of gene deletion mutants; plasmid pCA24N (Supplementary Figure 4) was used for gene expression and purification; and plasmid pZT102 was a reporter plasmid used for *lacZ*-fusion. The details for relevent strains and plasmids are listed in Supplementary Table 5. The gene-specific primers used for the construction of the recombinant plasmids are listed in Supplementary Table 6. Where appropriate, the *E. coli* culture medium was supplemented with 50 μg ml^–1^ kanamycin, 50 μg ml^–1^ chloramphenicol, and 100 μg ml^–1^ ampicillin.

### Construction of Gene Deletion Mutants, Complemented Strains, and Gene Overexpression Strains

We constructed the *E. coli* W3110 gene knockout mutants with the λ Red recombination system and verified mutants with junction PCR as previously described (Datsenko and Wanner, 2000). Double-knockout mutants were generated with the same procedure. We amplified the genes *mgrB*, *phoP*, *phoQ*, *phoPQ*, *pmrK*, and *tolC* genes from *E. coli* W3110 genomic DNA using gene specific primers (see Supplementary Table 6), and then cloned the genes into plasmids pCA24N. The recombinant plasmids were then transduced into either the deletion mutants of *E. coli* W3110 (to form the complemented strain) or the *E. coli* wild types (to form the gene overexpression strain). Plasmid pCA24N:*phoP* was transduced into *E. coli* BL21(DE3) for the overexpression and purification of the protein PhoP. The details for relevent strains and plasmids are listed in Supplementary Table 5.

### Screening for Genes Associated With TMP Resistance or Susceptibility

We cultured the *E. coli* Keio Knockout Collection (Baba et al., 2006) in 96 Deepwell Multiwell plates in Luria-Bertani (LB) medium (Difco) at 37°C until the OD_600_ was 0.6. We then diluted each well to 10^7^ colony-forming units (cfu) ml^–1^ (about OD_600_ of 0.01) with fresh LB containing either 0.1 μg ml^–1^ or 1 μg ml^–1^ TMP. Plates were statically incubated overnight (12 h) at 37°C. We measured the absorbance of each well at 600 nm with a Microplate Reader (BioTek, Winooski, VT, United States) to monitor the growth of all mutants.

### Drug Susceptibility Tests

Bacterial cells were grown to the mid-log phase (OD_600_ of ∼0.6), then diluted to 10^5^ cfu ml^–1^ in fresh LB. Next, ten-fold serial dilutions were plated onto LB agar solid plates containing different concentrations of TMP (0, 0.01, 0.02, 0.04, 0.08, 0.16, 0.32, 0.64, 1.28, and 2.56 μg ml^–1^). 10 μM IPTG was contained when the complementation and/or over-expression strains were tested. Cultures were incubated overnight at 37°C. The MIC (minimum inhibitory concentration) was defined as the lowest concentration of the TMP required to inhibit 99% of the colony-forming bacterial units. Each strain tested two times with at least three different clones.

### RNA-Seq

Total RNA was isolated with a RNeasy mini kit (Qiagen, Hilden, Germany). Library constructions were prepared using a TruSeq Stranded Total RNA Sample Preparation kit (Illumina, San Diego, CA, United States), and RNA was sequenced with an Illumina HiSeq 2500 at the Shanghai Biotechnology Corporation (Shanghai, China). The insert size of the purified libraries was confirmed with an Agilent 2100 bioanalyzer (Agilent Technologies, Santa Clara, CA, United States). Bowtie2 v2-2.0.5 was used to map the cleaned reads to the genome of *E. coli* str. K-12 substr. W3110 (downloaded from the National Center for Biotechnology Information)^1^. To estimate fold changes, HTSeq v2.1.1 was used with a reference annotation to generate values for fragments per kilobase of exon model per million mapped reads. Three biological replicates were used for RNA-seq, and *p*- and *q*-values were calculated. Differentially expressed genes were selected only if the false discovery rate *q*-value ≤ 0.05 and fold-change ≥ 2.

### Quantitative Real-Time PCR Assays

Total RNA was extracted and cDNA was synthesized with a ReverTra Ace quantitative polymerase chain reaction (qPCR) kit (TOYOBO, Osaka, Japan), following the manufacturer’s instructions. We used real-time PCR (RT-PCR) to quantify gene expression levels using a 7900 HT Sequence Detection System with ABI Power SYBR Green PCR Master Mix (ABI, Hudson, NH, United States). Primers specific to *phoQ*, *phoP*, and *tolC* were designed and synthesized by Sangon Biotech Co., Ltd. (Shanghai, China). Expression levels of *gapA* mRNA were normalized as an endogenous control. All gene-specific primers are listed in Supplementary Table 6.

### Protein Purification and Phosphorylation

Cultures of *E. coli* BL21 (DE3)/pCA24N:*phoP* were incubated at 37°C in LB broth medium supplied with corresponding antibiotics until the exponential phase. IPTG (isopropyl β-D-thiogalactoside) was added to produce a final concentration of 0.1 mM, and cultures were then incubated overnight with shaking at 16°C. Bacterial cells were harvested and then disrupted by sonication. The cell lysate was centrifuged at 10,000 × *g* for 30 min and the protein was isolated from the supernatant by nickel affinity chromatography. The purified His_6_-PhoP protein was dialyzed against 50 mM Tris–HCl buffer (pH 7.5). For EMSA assays, the His_6_-PhoP was phosphorylated as previously described (Tang et al., 2012) with modifications. Briefly, His_6_-PhoP was incubated in phosphorylation buffer (50 mM Tris–HCl [pH 7.5], 50 mM KCl, 10 mM MgCl_2_) containing 10 mM acetyl phosphate (Sigma-Aldrich) for 1 h at 37°C.

### Electrophoretic Mobility Shift Assay (EMSA)

The biotin-labeled probes containing the promoter regions of the gene *folA* were amplified using biotin-labeled primers (Supplementary Table 6). EMSA/Gel-Shift Kit (Beyotime, Shanghai, China) was used for the binding of probes and phosphorylated PhoP proteins. We resolved band shifts on 6% non-denaturing TBE (Tris-Boric acid-EDTA)-polyacrylamide gels and subjected the gels to electrophoresis at 80 V for 1 h at 4°C. DNA was transferred to a nylon membrane at 380 mA for 70 min, and ultraviolet cross-linked at 302 nm for 15 min. We subsequently developed membranes using the Chemiluminescent Biotin-labeled Nucleic Acid Detection Kit (Beyotime, Shanghai, China), following the manufacturer’s protocol. We detected signals using a Typhoon 9410 imager (GE Healthcare, Maple Grove, MN, United States).

### *lacZ*-Fusion Construction and β-Galactosidase Assays

The DNA fragments containing promoter regions (including original sequence and sequence with mutation sites) of the *folA* operon were amplified with *Bam*HI- and *Hin*dIII-included primers (see Supplementary Table 6). Amplified fragments were ligated with pZT102 reporter plasmids containing the *lacZ* operon (obtained from Prof. Shiyun Chen, Wuhan Institute of Virology, Chinese Academy of Sciences, China) to generate a series of *lacZ*-fused plasmids (Supplementary Table 5). The plasmids were then electroporated into *E. coli* strains (*E. coli* W3110, *E. coli* W3110 Δ*phoP*, *E. coli* W3110 Δ*mgrB*, and *E. coli* W3110 Δ*mgrB* Δ*phoP*). We cultured the recombinant strains at 37°C overnight, then diluted the cultures to 1% with fresh LB broth, grew them again at 37°C, and finally harvested them at an OD_600_ of ∼0.7. We washed cell pellets with PBS and assayed β-galactosidase activity as described by Miller (1992).

### DNase I Footprinting Assay

The promoter region of *folA* (−216 to −12) was amplified using FAM-labeled reverse primer (Supplementary Table 6). The purified DNA fragment (800 ng) was added to the reaction mixture (containing phosphorylated PhoP proteins) at 37°C for 30 min as described in EMSA. All of the mixtures were treated with DNase (1 unit, Fermentas China Co., Ltd., Shenzhen, China) at 37°C for 45 s. The results were analyzed with Applied Biosystems 37030XL DNA analyzer (manufactured by Tsingke Company, Wuhan, China).

### Gene Replacement

The promoter region of *folA* in the chromosome of *E. coli* W3110 Δ*mgrB* was replaced with the wild type promoter fragment, the deletion mutated promoter fragment (−166 to −147 deleted) respectively with linked Kanamycin resistance gene as a selection marker. The gene replacement mutants were constructed with the λ Red recombination system as previously described (Datsenko and Wanner, 2000). The mutants were verified by junction PCR and subsequent sequencing using primers that anneal to the genomic region outside the recombination locus.

## Data Availability Statement

The datasets presented in this study can be found in online repositories. The names of the repository/repositories and accession number(s) can be found below: The RNA sequencing reads was deposited in the Sequence Read Archive (SRA) repositories, and the accession number was PRJNA523715 [FEMS Microbiol Lett. 2019, 366(11):fnz123]. The primers used in qRT-PCR have been added into Supplementary Table 5.

## Author Contributions

HS, TL, JX, and JY have made major contributions to acquisition and analysis of data. SY, X-EZ, and ST have made contributions to interpretation of data. JG and J-YD have made substantial contributions to (i) conception and design, (ii) acquisition, analysis, and interpretation of data, and (iii) writing of the manuscript. All authors contributed to the article and approved the submitted version.

## Conflict of Interest

The authors declare that the research was conducted in the absence of any commercial or financial relationships that could be construed as a potential conflict of interest.

## Publisher’s Note

All claims expressed in this article are solely those of the authors and do not necessarily represent those of their affiliated organizations, or those of the publisher, the editors and the reviewers. Any product that may be evaluated in this article, or claim that may be made by its manufacturer, is not guaranteed or endorsed by the publisher.

## Footnotes

1
https://www.ncbi.nlm.nih.gov/genome/167?genome_assembly_id=61529
