# Supplementary figures and images for: MgrB Inactivation Confers Trimethoprim Resistance in Escherichia coli
Source: Front Microbiol. 2021 Jul 28;12:682205. doi: 10.3389/fmicb.2021.682205 (PMC8355897; doi:10.3389/fmicb.2021.682205)

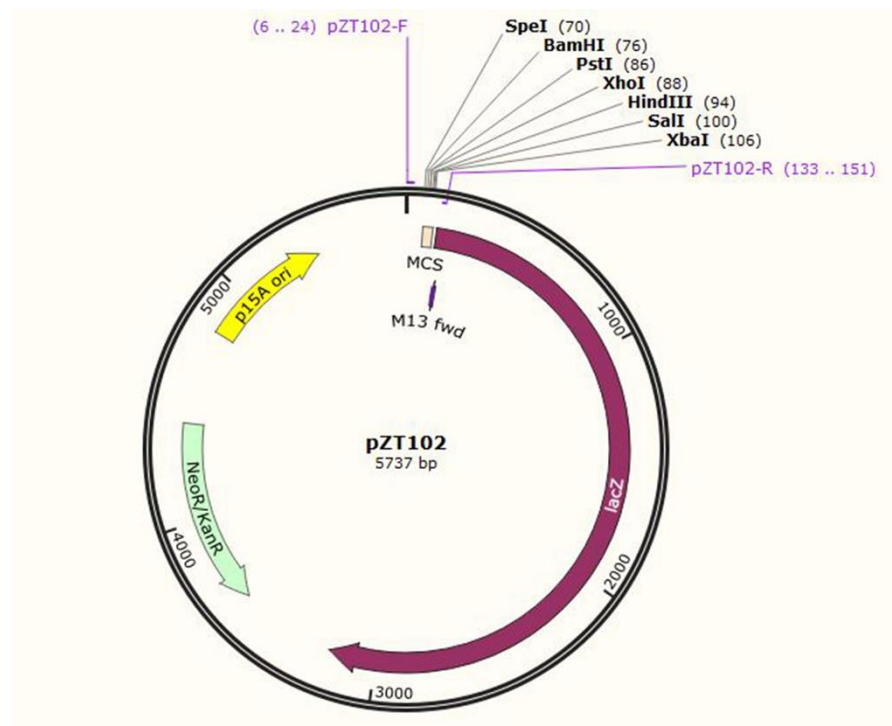

**Figure S1 The structural map of pZT102 plasmid vector**

Supplement: Supplementary file 1 [file Data_Sheet_1.PDF]

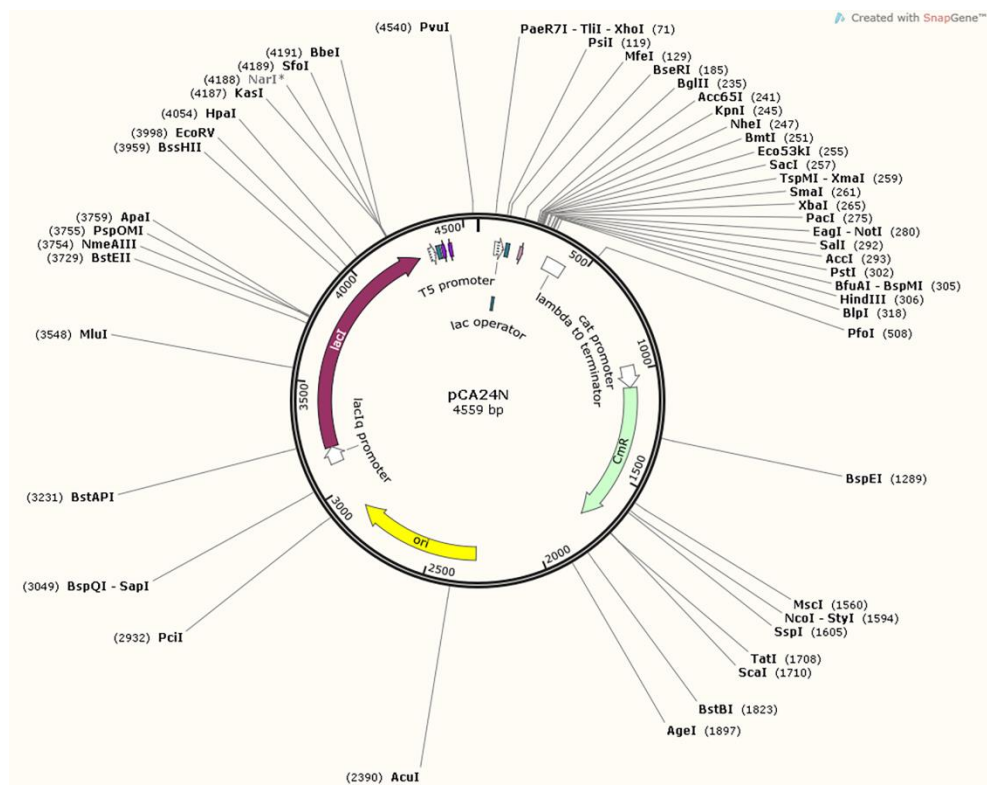

**Figure S4 The structural map of pCA24N plasmid vector**

Supplement: Supplementary file 4 [file Data_Sheet_4.PDF]
