# Supplementary material for: MgrB Inactivation Confers Trimethoprim Resistance in Escherichia coli
Source: Front Microbiol. 2021 Jul 28;12:682205. doi: 10.3389/fmicb.2021.682205 (PMC8355897; doi:10.3389/fmicb.2021.682205)
Supplement: Supplementary file 2 [file Data_Sheet_2.PDF]

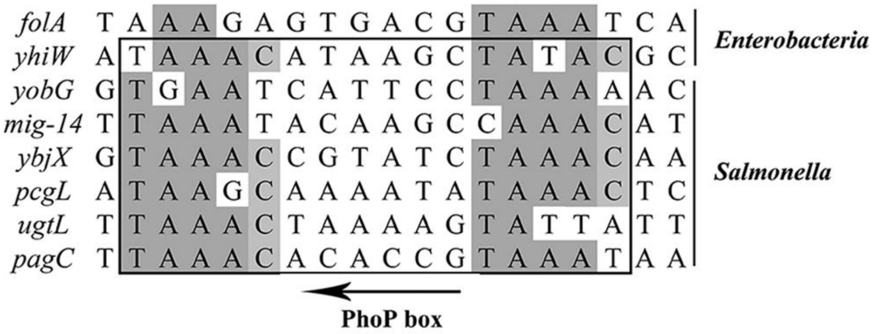

Figure S2 Alignment of the putative PhoP box of *folA* and seven "reverse PhoP boxes" with the similar architecture in *Enterobacteria* and *Salmonella*. The PhoP boxes are indicated with black box. The PhoP box orientation is indicated with black arrow.
