# Supplementary material for: MgrB Inactivation Confers Trimethoprim Resistance in Escherichia coli
Source: Front Microbiol. 2021 Jul 28;12:682205. doi: 10.3389/fmicb.2021.682205 (PMC8355897; doi:10.3389/fmicb.2021.682205)
Supplement: Supplementary file 3 [file Data_Sheet_3.PDF]

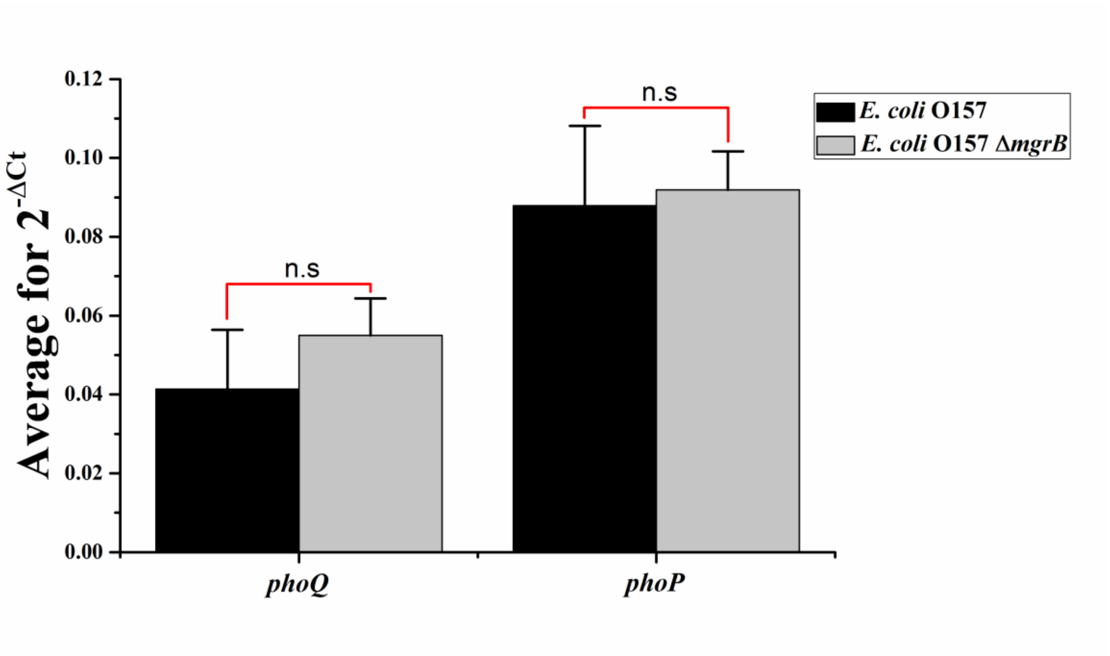

Figure S3 Average transcription levels of the genes *phoQ* and *phoP* in *E. coli* O157 (wild type) and *E. coli* O157 Δ*mgrB*, as measured with qRT-PCR. Expression levels of *gapA* was normalized as an endogenous control. Data represent mean ± SD from three independent experiments. n.s indicates no difference.
