# Supplementary material for: MgrB Inactivation Confers Trimethoprim Resistance in Escherichia coli
Source: Front Microbiol. 2021 Jul 28;12:682205. doi: 10.3389/fmicb.2021.682205 (PMC8355897; doi:10.3389/fmicb.2021.682205)
Supplement: Supplementary file 5 [file Table_1.DOCX]

**Table S1**. List of genes potentially associated with TMP susceptibility in *E. coli* K-12 BW25113. Cultures were incubated overnight at 37°C on LB.

| **Gene name** | **Growth of Gene deletion mutants in the presence of 0.1 μg ml^-1^ TMP (OD_600_)** | **Growth of Gene deletion mutants in the absence of TMP (OD_600_)** | **Growth of Gene deletion mutants in the presence of 1 μg ml^-1^ TMP (OD_600_)** |
| --- | --- | --- | --- |
| Wild type  *ybeB* | 0.641  0.763 | 0.723  0.945 | 0.06  0.742 |
| *mgrB* | 1.114 | 0.983 | 0.288 |
| *ymjC* | 1.084 | 0.676 | 0.264 |
| *yjjB* | / | 0.893 | 0.292 |
| *yjfO* | 0.743 | 1.083 | 0.224 |
| *fis* | 0.88 | 0.845 | 0.252 |
| *ymfG* | 0.93 | 1.08 | 0.278 |
| *leuD* | 0.86 | 0.823 | 0.322 |
| *nuoJ* | 0.607 | 0.568 | 0.216 |
| *tehB* | 0.878 | 1.12 | 0.271 |
| *mpaA* | 0.078 | 0.639 | 0.746 |
| *btuC* | 1.094 | 0.527 | 0.371 |
| *yeaX* | 1.088 | 0.671 | 0.3 |
| *cysB* | 0.502 | 0.694 | 0.241 |
| *yfbP* | 0.848 | 0.629 | 0.27 |
| *yhfK* | 1.085 | 0.656 | 0.269 |
| *ypfG* | 1.06 | 0.896 | 0.263 |
| *basS* | 0.958 | 0.593 | 0.22 |
| *sufS* | 0.966 | 0.917 | 0.238 |
| *torR* | 0.383 | 0.593 | 0.063 |
| *rcsC* | 0.415 | 0.744 | 0.075 |
| *rfaH* | 0.3 | 1.07 | 0.05 |
| *pgpA* | 0.384 | 0.677 | 0.054 |
| *hha* | 0.53 | 0.653 | 0.15 |
| *rfaP* | 0.372 | 0.85 | 0.061 |
| *ybiX* | 0.395 | 1 | 0.117 |
| *acrB* | 0.333 | 0.796 | 0.059 |
| *yceA* | 0.361 | 1.025 | 0.067 |
| *acrA* | 0.142 | 0.579 | 0.051 |
| *phoQ* | 0.413 | 0.591 | 0.12 |
| *phoP* | 0.4 | 0.58 | 0.11 |
