# Supplementary material for: MgrB Inactivation Confers Trimethoprim Resistance in Escherichia coli
Source: Front Microbiol. 2021 Jul 28;12:682205. doi: 10.3389/fmicb.2021.682205 (PMC8355897; doi:10.3389/fmicb.2021.682205)
Supplement: Supplementary file 7 [file Table_3.docx]

**Table S3**. Fluorescence signal value of footprinting assay in segment 2

| Position | **134** | **138** | **139** | **140** | **143** | **144** | **148** | **152** | **153** | **155** | **158** | **160** | **161** | **163.2** | **164** | **165** |
| --- | --- | --- | --- | --- | --- | --- | --- | --- | --- | --- | --- | --- | --- | --- | --- | --- |
| With PhoP | 2291 | 1471 | 791 | 1250 | 0 | 4229 | 2540 | 2952 | 899 | 1478 | 1536 | 0 | 1496 | 0 | 1437 | 2147 |
| Without PhoP | 3579 | 1945 | 1123 | 1674 | 1992 | 6811 | 4039 | 4984 | 1558 | 2768 | 3001 | 1111 | 2596 | 1124 | 2897 | 4187 |
| With PhoP/Without PhoP | 0.64 | 0.76 | 0.70 | 0.75 | 0 | 0.62 | 0.63 | 0.59 | 0.58 | 0.53 | 0.51 | 0 | 0.58 | 0 | 0.50 | 0.51 |
