# Supplementary material for: MgrB Inactivation Confers Trimethoprim Resistance in Escherichia coli
Source: Front Microbiol. 2021 Jul 28;12:682205. doi: 10.3389/fmicb.2021.682205 (PMC8355897; doi:10.3389/fmicb.2021.682205)
Supplement: Supplementary file 8 [file Table_4.DOCX]

**Table S4**. MICs (μg ml^-1^) to TMP in different strains of *E. coli* EHEC O157:H7. Cultures were incubated overnight at 37°C on LB.

| **Strain** | **MIC to TMP** |
| --- | --- |
| O157:H7 | 0.64 |
| O157:H7 △*mgrB* | 0.32 |
