# Supplementary material for: MgrB Inactivation Confers Trimethoprim Resistance in Escherichia coli
Source: Front Microbiol. 2021 Jul 28;12:682205. doi: 10.3389/fmicb.2021.682205 (PMC8355897; doi:10.3389/fmicb.2021.682205)
Supplement: Supplementary file 9 [file Table_5.docx]

**Table S5.** Plasmids, strains used in this study

| **Category** | **Name** | **Relevant Features** | | **Source** |
| --- | --- | --- | --- | --- |
| **Library** | *E. coli* Keio Knockout Collection | *E. coli* BW25113 | | (Baba et al., 2006) |
| **Plasmids** |  |  | |  |
|  | pKD4 | Template plasmids containing a kanamycin resistance gene flanked by FRT sites, Amp^R^, Km^R^ | | (Datsenko and Wanner, 2000) |
|  | pKD46 | Red recombinase expression plasmids, Amp^R^ | | (Datsenko and Wanner, 2000) |
|  | pCP20 | FLP helper plasmid, Amp^R^, Cm^R^ | | (Datsenko and Wanner, 2000) |
|  | pCA24N | Cm^R^ | Presented by Prof. Shengce Tao | |
|  | pZT102 | Kan^R^ | Presented by Prof. Shiyun Chen | |
|  | pCA24N::*phoP* | *phoP* expression plasmids, Cm^R^ | | This study |
|  | pZT102::*folA*p-257 | *lacZ*-fusion plasmid containing 257 bp *folA* promoter region, Kan^R^ | | This study |
|  | pZT102::*folA*p-152 | *lacZ*-fusion plasmid containing152 bp *folA* promoter, Kan^R^ | | This study |
|  | pZT102::*folA*p-99 | *lacZ*-fusion plasmid containing 99 bp *folA* promoter, Kan^R^ | | This study |
|  | pZT102::*folA*p-66 | *lacZ*-fusion plasmid containing 66 bp *folA* promoter, Kan^R^ | | This study |
|  | pZT102::*folA*p-191 | *lacZ*-fusion plasmid containing 191 bp *folA* promoter, Kan^R^ | | This study |
|  | pZT102::*folA*p-191mut1 | *lacZ*-fusion plasmid containing 191 bp *folA* promoter with site-mutation A (−162) A (−163) to GG, Kan^R^ | | This study |
|  | pZT102::*folA*p-191mut2 | *lacZ*-fusion plasmid containing 191 bp *folA* promoter with site-mutation A (−150) A (−151) to GG, Kan^R^ | | This study |
|  | pCA24N:: *mgrB* | *mgrB* expression plasmids, Cm^R^ | | This study |
|  | pCA24N::*phoP* | *phoP* expression plasmids, Cm^R^ | | This study |
|  | pCA24N:: *phoQ* | *phoQ* expression plasmids, Cm^R^ | | This study |
|  | pCA24N:: *phoPQ* | *phoPQ* expression plasmids, Cm^R^ | | This study |
|  | pCA24N:: *pmrK* | *pmrK* expression plasmids, Cm^R^ | | This study |
|  | pCA24N:: *tolC* | *tolC* expression plasmids, Cm^R^ | | This study |
|  | pCA24N:: *ymjC* | expression plasmids, Cm^R^ | | This study |
|  | pCA24N:: *tehB* | *tehB* expression plasmids, Cm^R^ | | This study |
|  | pCA24N:: *cysB* | *cysB* expression plasmids, Cm^R^ | | This study |
|  | pCA24N:: *rfaH* | *rfaH* expression plasmids, Cm^R^ | | This study |
|  | pCA24N:: *acrB* | *acrB* expression plasmids, Cm^R^ | | This study |
|  | pCA24N:: *acrA* | *acrA* expression plasmids, Cm^R^ | | This study |
|  | pCA24N:: *torR* | *torR* expression plasmids, Cm^R^ | | This study |
|  | pCA24N:: *rcsC* | *rcsC* expression plasmids, Cm^R^ | | This study |
|  | pCA24N:: *pgpA* | *pgpA* expression plasmids, Cm^R^ | | This study |
|  | pCA24N:: *yhfK* | *yhfK* expression plasmids, Cm^R^ | | This study |
|  | pCA24N:: *ybeB* | *ybeB* expression plasmids, Cm^R^ | | This study |
|  | pCA24N:: *leuD* | *leuD* expression plasmids, Cm^R^ | | This study |
|  | pCA24N:: *ypfG* | *ypfG* expression plasmids, Cm^R^ | | This study |
|  | pCA24N:: *btuC* | *btuC* expression plasmids, Cm^R^ | | This study |
|  | pCA24N:: *yjjB* | *yjjB* expression plasmids, Cm^R^ | | This study |
|  | pCA24N:: *yjfO* | *yjfO* expression plasmids, Cm^R^ | | This study |
|  | pCA24N:: *yeaX* | *yeaX* expression plasmids, Cm^R^ | | This study |
|  | pCA24N:: *nuoJ* | *nuoJ* expression plasmids, Cm^R^ | | This study |
|  | pCA24N:: *bass* | *bass* expression plasmids, Cm^R^ | | This study |
|  | pCA24N:: *fis* | *fis* expression plasmids, Cm^R^ | | This study |
|  | pCA24N:: *sufS* | *sufS* expression plasmids, Cm^R^ | | This study |
|  | pCA24N:: *ymfG* | *ymfG* expression plasmids, Cm^R^ | | This study |
|  | pCA24N:: *rfaP* | *rfaP* expression plasmids, Cm^R^ | | This study |
|  | pCA24N:: *yceA* | *yceA* expression plasmids, Cm^R^ | | This study |
|  | pCA24N:: *flgE* | *flgE* expression plasmids, Cm^R^ | | This study |
|  | pCA24N:: *ybiX* | *ybiX* expression plasmids, Cm^R^ | | This study |
|  | pCA24N:: *yfbP* | *yfbP* expression plasmids, Cm^R^ | | This study |
|  | pCA24N:: *mpaA* | *mpaA* expression plasmids, Cm^R^ | | This study |
|  | pCA24N:: *ybaJ* | *ybaJ* expression plasmids, Cm^R^ | | This study |
|  | pCA24N:: *hha* | *hha* expression plasmids, Cm^R^ | | This study |
|  | pCA24N:: *folA* | *folA* expression plasmids, Cm^R^ | | This study |
| **Strains** |  |  | |  |
|  | *E. coli* W3110 | F^-^ λ^-^ rph-1 INV(rrnD, rrnE) | | Lab Stock |
|  | *E. coli* BL21(DE3) | *E. coli* str. B F^–^ *ompT* *gal* *dcm* *lon* *hsdS_B_*(*r_B_*^–^*m_B_*^–^) λ(DE3 [*lacI* *lacUV5*-*T7p07* *ind1* *sam7* *nin5*]) [*malB*^+^]_K-12_(λ^S^) | | Lab Stock |
|  | *E. coli* BL21(DE3) pCA24N::*phoP* | *E. coli* BL21(DE3) transformed with pCA24N::*phoP* | | This study |
|  | *E. coli* W3110 Δ*mgrB* | *mgrB* gene deleted in *E. coli* W3110 | | This study |
|  | *E. coli* W3110 Δ*phoP* | *phoP* gene deleted in *E. coli* W3110 | | This study |
|  | *E. coli* W3110 Δ*phoQ* | *phoQ* gene deleted in *E. coli* W3110 | | This study |
|  | *E. coli* W3110 Δ*mgrB* Δ*phoQ* | *mgrB* and *phoQ* genes deleted in *E. coli* W3110 | | This study |
|  | *E. coli* W3110 Δ*mgrB* Δ*phoP* | *mgrB* and *phoP* genes deleted in *E. coli* W3110 | | This study |
|  | *E. coli* W3110 Δ*tolC* | *tolC* gene deleted in *E. coli* W3110 | | This study |
|  | *E. coli* W3110 Δ*pmrK* | *pmrK* gene deleted in *E. coli* W3110 | | This study |
|  | *E. coli* W3110::pZT102 | *E. coli* W3110 transformed with pZT102 | | This study |
|  | *E. coli* W3110 pZT102::*folA*p-257 | *E. coli* W3110 transformed with pZT102::*folA*p-257 | | This study |
|  | *E. coli* W3110 pZT102::*folA*p-152 | *E. coli* W3110 transformed with pZT102::*folA*p-152 | | This study |
|  | *E. coli* W3110 pZT102::*folA*p-99 | *E. coli* W3110 transformed with pZT102::*folA*p-99 | | This study |
|  | *E. coli* W3110 pZT102::*folA*p-66 | *E. coli* W3110 transformed with pZT102::*folA*p-66 | | This study |
|  | *E. coli* W3110 pZT102::*folA*p-191 | *E. coli* W3110 transformed with pZT102::*folA*p-191 | | This study |
|  | *E. coli* W3110 pZT102::*folA*p-191mut1 | *E. coli* W3110 transformed with pZT102::*folA*p-191mut1 | | This study |
|  | *E. coli* W3110 pZT102::*folA*p-191mut2 | *E. coli* W3110 transformed with pZT102::*folA*p-191mut2 | | This study |
|  | *E. coli* W3110 Δ*phoP*  pZT102::*folA*p-257 | *E. coli* W3110 Δ*phoP* transformed with pZT102::*folA*p-257 | | This study |
|  | *E. coli* W3110 Δ*phoP*  pZT102::*folA*p-191 | *E. coli* W3110 Δ*phoP* transformed with pZT102::*folA*p-191 | | This study |
|  | *E. coli* W3110 Δ*phoP*  pZT102::*folA*p-191mut1 | *E. coli* W3110 Δ*phoP* transformed with pZT102::*folA*p-191mut1 | | This study |
|  | *E. coli* W3110 Δ*phoP*  pZT102::*folA*p-191mut2 | *E. coli* W3110 Δ*phoP* transformed with pZT102::*folA*p-191mut2 | | This study |
|  | *E. coli* W3110 Δ*mgrB*  pZT102::*folA*p-257 | *E. coli* W3110 Δ*mgrB* transformed with pZT102::*folA*p-257 | | This study |
|  | *E. coli* W3110 Δ*mgrB* Δ*phoP* pZT102::*folA*p-257 | *E. coli* W3110 Δ*mgrB* Δ*phoP* transformed with pZT102::*folA*p-257 | | This study |
|  | *E. coli* W3110 pCA24N | *E. coli* W3110 transformed with pCA24N | | This study |
|  | *E. coli* W3110 Δ*mgrB* pCA24N | *E. coli* W3110 Δ*mgrB* transformed with pCA24N | | This study |
|  | *E. coli* W3110 Δ*phoP* pCA24N | *E. coli* W3110 Δ*phoP* transformed with pCA24N | | This study |
|  | *E. coli* W3110 Δ*phoQ* pCA24N | *E. coli* W3110 Δ*phoQ* transformed with pCA24N | | This study |
|  | *E. coli* W3110 Δ*mgrB* Δ*phoQ* pCA24N | *E. coli* W3110 Δ*mgrB* Δ*phoQ* transformed with pCA24N | | This study |
|  | *E. coli* W3110 Δ*mgrB* Δ*phoP* pCA24N | *E. coli* W3110 Δ*mgrB* Δ*phoP* transformed with pCA24N | | This study |
|  | *E. coli* W3110 Δ*tolC* pCA24N | *E. coli* W3110 Δ*tolC* transformed with pCA24N | | This study |
|  | *E. coli* W3110 Δ*pmrK* pCA24N | *E. coli* W3110 Δ*pmrK* transformed with pCA24N | | This study |
|  | E. coli W3110 ΔphoP pCA24N::phoP | complemented strain of *E. coli* W3110 Δ*phoP* | | This study |
|  | *E. coli* W3110 Δ*phoQ* pCA24N:: *phoQ* | complemented strain of *E. coli* W3110 Δ*phoQ* | | This study |
|  | *E. coli* W3110 Δ*pmrK*  pCA24N:: *pmrK* | complemented strain of *E. coli* W3110 Δ*pmrK* | | This study |
|  | *E. coli* W3110 Δ*tolC* pCA24N:: *tolC* | complemented strain of *E. coli* W3110 Δ*tolC* | | This study |
|  | *E. coli* W3110 pCA24N::*phoPQ* | *phoQ* and *phoP* overexpressed in *E. coli* W3110 | | This study |
|  | *E. coli* W3110 pCA24N:: *mgrB* | *mgrB* overexpressed in *E. coli* W3110 | | This study |
|  | *E. coli* W3110 pCA24N::*phoP* | *phoP* overexpressed in *E. coli* W3110 | | This study |
|  | *E. coli* W3110 pCA24N:: *phoQ* | *phoQ* overexpressed in *E. coli* W3110 | | This study |
|  | *E. coli* W3110 pCA24N:: *pmrK* | *pmrK* overexpressed in *E. coli* W3110 | | This study |
|  | *E. coli* W3110 pCA24N:: *tolC* | *tolC* overexpressed in *E. coli* W3110 | | This study |
|  | *E. coli* W3110 Δ*ymjC* pCA24N | *ymjC* gene deleted mutants transformed with pCA24N | | This study |
|  | *E. coli* W3110 Δ*tehB* pCA24N | *tehB* gene deleted mutants transformed with pCA24N | | This study |
|  | *E. coli* W3110 Δ*cysB* pCA24N | *cysB* gene deleted mutants transformed with pCA24N | | This study |
|  | *E. coli* W3110 Δ*rfaH* pCA24N | *rfaH* gene deleted mutants transformed with pCA24N | | This study |
|  | *E. coli* W3110 Δ*acrB* pCA24N | *acrB* gene deleted mutants transformed with pCA24N | | This study |
|  | *E. coli* W3110 Δ*acrA* pCA24N | *acrA* gene deleted mutants transformed with pCA24N | | This study |
|  | *E. coli* W3110 Δ*torR* pCA24N | *torR* gene deleted mutants transformed with pCA24N | | This study |
|  | *E. coli* W3110 Δ*rcsC* pCA24N | *rcsC* gene deleted mutants transformed with pCA24N | | This study |
|  | *E. coli* W3110 Δ*pgpA* pCA24N | *pgpA* gene deleted mutants transformed with pCA24N | | This study |
|  | *E. coli* W3110 Δ*yhfK* pCA24N | *yhfK* gene deleted mutants transformed with pCA24N | | This study |
|  | *E. coli* W3110 Δ*ybeB* pCA24N | *ybeB* gene deleted mutants transformed with pCA24N | | This study |
|  | *E. coli* W3110 Δ*leuD* pCA24N | *leuD* gene deleted mutants transformed with pCA24N | | This study |
|  | *E. coli* W3110 Δ*ypfG* pCA24N | *ypfG* gene deleted mutants transformed with pCA24N | | This study |
|  | *E. coli* W3110 Δ*btuC* pCA24N | *btuC* gene deleted mutants transformed with pCA24N | | This study |
|  | *E. coli* W3110 Δ*yjjB* pCA24N | *yjjB* gene deleted mutants transformed with pCA24N | | This study |
|  | *E. coli* W3110 Δ*yjfO* pCA24N | *yjfO* gene deleted mutants transformed with pCA24N | | This study |
|  | *E. coli* W3110 Δ*yeaX* pCA24N | *yeaX* gene deleted mutants transformed with pCA24N | | This study |
|  | *E. coli* W3110 Δ*nuoJ* pCA24N | *nuoJ* gene deleted mutants transformed with pCA24N | | This study |
|  | *E. coli* W3110 Δ*bass* pCA24N | *bass* gene deleted mutants transformed with pCA24N | | This study |
|  | *E. coli* W3110 Δ*fis* pCA24N | *fis* gene deleted mutants transformed with pCA24N | | This study |
|  | *E. coli* W3110 Δ*sufS* pCA24N | *sufS* gene deleted mutants transformed with pCA24N | | This study |
|  | *E. coli* W3110 Δ*ymfG* pCA24N | *ymfG* gene deleted mutants transformed with pCA24N | | This study |
|  | *E. coli* W3110 Δ*rfaP* pCA24N | *rfaP* gene deleted mutants transformed with pCA24N | | This study |
|  | *E. coli* W3110 Δ*yceA* pCA24N | *yceA* gene deleted mutants transformed with pCA24N | | This study |
|  | *E. coli* W3110 Δ*flgE* pCA24N | *flgE* gene deleted mutants transformed with pCA24N | | This study |
|  | *E. coli* W3110 Δ*ybiX* pCA24N | *ybiX* gene deleted mutants transformed with pCA24N | | This study |
|  | *E. coli* W3110 Δ*yfbP* pCA24N | *yfbP* gene deleted mutants transformed with pCA24N | | This study |
|  | *E. coli* W3110 Δ*mpaA* pCA24N | *mpaA* gene deleted mutants transformed with pCA24N | | This study |
|  | *E. coli* W3110 Δ*ybaJ* pCA24N | *ybaJ* gene deleted mutants transformed with pCA24N | | This study |
|  | *E. coli* W3110 Δ*hha* pCA24N | *hha* gene deleted mutants transformed with pCA24N | | This study |
|  | *E. coli* W3110 Δ*ymjC* pCA24N::*ymjC* | complemented strain of *E. coli* W3110 Δ*ymjC* | | This study |
|  | *E. coli* W3110 Δ*tehB* pCA24N::*tehB* | complemented strain of *E. coli* W3110 Δ*tehB* | | This study |
|  | *E. coli* W3110 Δ*cysB* pCA24N::*cysB* | complemented strain of *E. coli* W3110 Δ*cysB* | | This study |
|  | *E. coli* W3110 Δ*rfaH* pCA24N::*rfaH* | complemented strain of *E. coli* W3110 Δ*rfaH* | | This study |
|  | *E. coli* W3110 Δ*acrB* pCA24N::*acrB* | complemented strain of *E. coli* W3110 Δ*acrB* | | This study |
|  | *E. coli* W3110 Δ*acrA* pCA24N::*acrA* | complemented strain of *E. coli* W3110 Δ*acrA* | | This study |
|  | *E. coli* W3110 Δ*torR* pCA24N::*torR* | complemented strain of *E. coli* W3110 Δ*torR* | | This study |
|  | *E. coli* W3110 Δ*rcsC* pCA24N::*rcsC* | complemented strain of *E. coli* W3110 Δ*rcsC* | | This study |
|  | *E. coli* W3110 Δ*pgpA* pCA24N::*pgpA* | complemented strain of *E. coli* W3110 Δ*pgpA* | | This study |
|  | *E. coli* W3110 Δ*yhfK* pCA24N::*yhfK* | complemented strain of *E. coli* W3110 Δ*yhfK* | | This study |
|  | *E. coli* W3110 Δ*ybeB* pCA24N::*ybeB* | complemented strain of *E. coli* W3110 Δ*ybeB* | | This study |
|  | *E. coli* W3110 Δ*leuD* pCA24N::*leuD* | complemented strain of *E. coli* W3110 Δ*leuD* | | This study |
|  | *E. coli* W3110 Δ*ypfG* pCA24N::*ypfG* | complemented strain of *E. coli* W3110 Δ*ypfG* | | This study |
|  | *E. coli* W3110 Δ*btuC* pCA24N::*btuC* | complemented strain of *E. coli* W3110 Δ*btuC* | | This study |
|  | *E. coli* W3110 Δ*yjjB* pCA24N::*yjjB* | complemented strain of *E. coli* W3110 Δ*yjjB* | | This study |
|  | *E. coli* W3110 Δ*yjfO* pCA24N::*yjfO* | complemented strain of *E. coli* W3110 Δ*yjfO* | | This study |
|  | *E. coli* W3110 Δ*yeaX* pCA24N::*yeaX* | complemented strain of *E. coli* W3110 Δ*yeaX* | | This study |
|  | *E. coli* W3110 Δ*nuoJ* pCA24N::*nuoJ* | complemented strain of *E. coli* W3110 Δ*nuoJ* | | This study |
|  | *E. coli* W3110 Δ*bass* pCA24N::*bass* | complemented strain of *E. coli* W3110 Δ*bass* | | This study |
|  | *E. coli* W3110 Δ*fis* pCA24N::*fis* | complemented strain of *E. coli* W3110 Δ*fis* | | This study |
|  | *E. coli* W3110 Δ*sufS* pCA24N::*sufS* | complemented strain of *E. coli* W3110 Δ*sufS* | | This study |
|  | *E. coli* W3110 Δ*ymfG* pCA24N::*ymfG* | complemented strain of *E. coli* W3110 Δ*ymfG* | | This study |
|  | *E. coli* W3110 Δ*rfaP* pCA24N::*rfaP* | complemented strain of *E. coli* W3110 Δ*rfaP* | | This study |
|  | *E. coli* W3110 Δ*yceA* pCA24N::*yceA* | complemented strain of *E. coli* W3110 Δ*yceA* | | This study |
|  | *E. coli* W3110 Δ*flgE* pCA24N::*flgE* | complemented strain of *E. coli* W3110 Δ*flgE* | | This study |
|  | *E. coli* W3110 Δ*ybiX* pCA24N::*ybiX* | complemented strain of *E. coli* W3110 Δ*ybiX* | | This study |
|  | *E. coli* W3110 Δ*yfbP* pCA24N::*yfbP* | complemented strain of *E. coli* W3110 Δ*yfbP* | | This study |
|  | *E. coli* W3110 Δ*mpaA* pCA24N::*mpaA* | complemented strain of *E. coli* W3110 Δ*mpaA* | | This study |
|  | *E. coli* W3110 Δ*ybaJ* pCA24N::*ybaJ* | complemented strain of *E. coli* W3110 Δ*ybaJ* | | This study |
|  | *E. coli* W3110 Δ*hha* pCA24N::*hha* | complemented strain of *E. coli* W3110 Δ*hha* | | This study |
|  | *E. coli* W3110 pCA24N::*ymjC* | *ymjC* overexpressed in *E. coli* W3110 | | This study |
|  | *E. coli* W3110 pCA24N::*tehB* | *tehB* overexpressed in *E. coli* W3110 | | This study |
|  | *E. coli* W3110 pCA24N::*cysB* | *cysB* overexpressed in *E. coli* W3110 | | This study |
|  | *E. coli* W3110 pCA24N::*rfaH* | *rfaH* overexpressed in *E. coli* W3110 | | This study |
|  | *E. coli* W3110 pCA24N::*acrB* | *acrB* overexpressed in *E. coli* W3110 | | This study |
|  | *E. coli* W3110 pCA24N::*acrA* | *acrA* overexpressed in *E. coli* W3110 | | This study |
|  | *E. coli* W3110 pCA24N::*torR* | *torR* overexpressed in *E. coli* W3110 | | This study |
|  | *E. coli* W3110 pCA24N::*rcsC* | *rcsC* overexpressed in *E. coli* W3110 | | This study |
|  | *E. coli* W3110 pCA24N::*pgpA* | *pgpA* overexpressed in *E. coli* W3110 | | This study |
|  | *E. coli* W3110 pCA24N::*yhfK* | *yhfK* overexpressed in *E. coli* W3110 | | This study |
|  | *E. coli* W3110 pCA24N::*ybeB* | *ybeB* overexpressed in *E. coli* W3110 | | This study |
|  | *E. coli* W3110 pCA24N::*leuD* | *leuD* overexpressed in *E. coli* W3110 | | This study |
|  | *E. coli* W3110 pCA24N::*ypfG* | *ypfG* overexpressed in *E. coli* W3110 | | This study |
|  | *E. coli* W3110 pCA24N::*btuC* | *btuC* overexpressed in *E. coli* W3110 | | This study |
|  | *E. coli* W3110 pCA24N::*yjjB* | *yjjB* overexpressed in *E. coli* W3110 | | This study |
|  | *E. coli* W3110 pCA24N::*yjfO* | *yjfO* overexpressed in *E. coli* W3110 | | This study |
|  | *E. coli* W3110 pCA24N::*yeaX* | *yeaX* overexpressed in *E. coli* W3110 | | This study |
|  | *E. coli* W3110 pCA24N::*nuoJ* | *nuoJ* overexpressed in *E. coli* W3110 | | This study |
|  | *E. coli* W3110 pCA24N::*bass* | *bass* overexpressed in *E. coli* W3110 | | This study |
|  | *E. coli* W3110 pCA24N::*fis* | *fis* overexpressed in *E. coli* W3110 | | This study |
|  | *E. coli* W3110 pCA24N::*sufS* | *sufS* overexpressed in *E. coli* W3110 | | This study |
|  | *E. coli* W3110 pCA24N::*ymfG* | *ymfG* overexpressed in *E. coli* W3110 | | This study |
|  | *E. coli* W3110 pCA24N::*rfaP* | *rfaP* overexpressed in *E. coli* W3110 | | This study |
|  | *E. coli* W3110 pCA24N::*yceA* | *yceA* overexpressed in *E. coli* W3110 | | This study |
|  | *E. coli* W3110 pCA24N::*flgE* | *flgE* overexpressed in *E. coli* W3110 | | This study |
|  | *E. coli* W3110 pCA24N::*ybiX* | *ybiX* overexpressed in *E. coli* W3110 | | This study |
|  | *E. coli* W3110 pCA24N::*yfbP* | *yfbP* overexpressed in *E. coli* W3110 | | This study |
|  | *E. coli* W3110 pCA24N::*mpaA* | *mpaA* overexpressed in *E. coli* W3110 | | This study |
|  | *E. coli* W3110 pCA24N::*ybaJ* | *ybaJ* overexpressed in *E. coli* W3110 | | This study |
|  | *E. coli* W3110 pCA24N::*hha* | *hha* overexpressed in *E. coli* W3110 | | This study |

Baba, T., Ara, T., Hasegawa, M., Takai, Y., Okumura, Y., Baba, M., et al. (2006). Construction of Escherichia coli K-12 in-frame, single-gene knockout mutants: the Keio collection. *Mol Syst Biol.* 2**,** 2006 0008. doi: 10.1038/msb4100050

Datsenko, K.A., and Wanner, B.L. (2000). One-step inactivation of chromosomal genes in Escherichia coli K-12 using PCR products. *Proc Natl Acad Sci U S A.* 97**,** 6640-6645. doi: 10.1073/pnas.120163297
