# Supplementary material for: MgrB Inactivation Confers Trimethoprim Resistance in Escherichia coli
Source: Front Microbiol. 2021 Jul 28;12:682205. doi: 10.3389/fmicb.2021.682205 (PMC8355897; doi:10.3389/fmicb.2021.682205)
Supplement: Supplementary file 10 [file Table_6.docx]

**Table S6.** Primers used in this study

| **Primers** | **Sequence (5’-3’)** |
| --- | --- |
|  | **Primers used in construction of gene deletion mutants, complementary strains, and gene overexpression strains** |
| *ybeB*-Ex-F | AATTAGATCTTTGCAGGGTAAAGCACTCCAGGATT |
| *ybeB*-Ex-R | AATTTCTAGATTAACTCCAGAGTTTTTCCAGTTCA |
| *ybeB*-Ko-F | TTGCAGGGTAAAGCACTCCAGGATTTTGTTATCGACAAAATTGATGACCTCTATATGAATATCCTCCTTAG |
| *ybeB*-Ko-R | TTAACTCCAGAGTTTTTCCAGTTCATACAGGCGACGGCTCTCTTCCTGCATATGTAGGCTGGAGCTGCTTCG |
| *ybeB*-Id-F | GCGTCCTCAATAGCCGTGCCATCGGG |
| *ybeB*-Id-R | ACTGACGTCGCGACCATCCAGCTTCC |
| *leuD*-Ex-F | AATTAGATCTATGGCAGAGAAATTTATCAAACACA |
| *leuD*-Ex-R | AATTTCTAGATTAATTCATAAACGCAGGTTGTTTT |
| *leuD*-Ko-F | ATGGCAGAGAAATTTATCAAACACACAGGCCTGGTGGTTCCGCTGGATGCCTATATGAATATCCTCCTTAG |
| *leuD*-Ko-R | TTAATTCATAAACGCAGGTTGTTTTGCTTCATAAGCGGCAATGGCGTCGTTATGTAGGCTGGAGCTGCTTCG |
| *leuD*-Id-F | TGCAGGCACTGGTGGTTC |
| *leuD*-Id-R | CTGCAACTTTTTAACGCTGTATTT |
| *mgrB*-Ex-F | AATTAGATCTGTGAAAAAGTTTCGATGGGTCGTTC |
| *mgrB*-Ex-R | AATTTCTAGATAACCACGGGATAAACTGGTTAATG |
| *mgrB*-Ko-F | GTGAAAAAGTTTCGATGGGTCGTTCTGGTTGTCGTGGTGTTGGCTTGCTTCTATATGAATATCCTCCTTAG |
| *mgrB*-Ko-R | TCACCACGGGATAAACTGGTTAATGGCACAAATTCCGCTGAAAAATTGTATATGTAGGCTGGAGCTGCTTCG |
| *mgrB*-Id-F | TGCCTCTGGCAGTCAGATAGGTACA |
| *mgrB*-Id-R | TCTTTTTATCTGCTTTCTCTGGTTC |
| *ypfG*-Ex-F | AATTAGATCTATGCGCTATCGCATTTTCC |
| *ypfG*-Ex-R | AATTTCTAGATTAACGGGTGATCCACAACG |
| *ypfG*-Ko-F | ATGCGCTATCGCATTTTCCTTCTCTTTTTTTTCGCTTTGTTGCCGACGTCCTATATGAATATCCTCCTTAG |
| *ypfG*-Ko-R | TTAACGGGTGATCCACAACGTGGGCCAGGCGTCTGGCCCATGCCAGTTATTATGTAGGCTGGAGCTGCTTCG |
| *ypfG*-Id-F | CGAATACAGTGACAATCAGC |
| *ypfG*-Id-R | TGCCTTCTAACGTTGCGG |
| *btuC*-Ex-F | AATTGGTACCATGCTGACACTTGCCCGC |
| *btuC*-Ex-R | AATTTCTAGACTAACGTCCTGCTTTTAACAA |
| *btuC*-Ko-F | ATGCTGACACTTGCCCGCCAACAACAGCGACAAAATATTCGCTGGTTATTCTATATGAATATCCTCCTTAG |
| *btuC-Ko-R* | CTAACGTCCTGCTTTTAACAATAACCAGATAAACACCGGCGCACCTAACGTATGTAGGCTGGAGCTGCTTCG |
| *btuC*-Id-F | TCCGTCGCGCTCTGGAAAAC |
| *btuC*-Id-R | TAATCTCTTCATCGCTGCCCG |
| *ymjC*-Ex-F | AATTAGATCTATGAAAAATGTACTGATTCTTGGTG |
| *ymjC*-Ex-R | AATTTCTAGATAATCGATCTTGTATCACTTTTTTG |
| *ymjC*-Ko-F | ATGAAAAATGTACTGATTCTTGGTGCTGGCGGTCAGATAGCCCGCCATGTCTATATGAATATCCTCCTTAG |
| *ymjC*-Ko-R | TCATCGATCTTGTATCACTTTTTTGCCGGAAGTTGTTTGCATTTTATTAGTATGTAGGCTGGAGCTGCTTCG |
| *ymjC*-Id-F | ATGATGCTTACCATATGGCGGAAG |
| *ymjC*-Id-R | TGACGGCGCAAAGAAGGTGT |
| *yjjB*-Ex-F | AATTAGATCTATGATCTTGATGACCAGCGGGTTG |
| *yjjB*-Ex-R | AATTTCTAGATTATACGCGAGGGCGCTTGC |
| *yjjB*-Ko-F | ATGATCTTGATGACCAGCGGGTTGAATATTGAGTGGTCAACCTTTATGGCCTATATGAATATCCTCCTTAG |
| *yjjB*-Ko-R | TTATACGCGAGGGCGCTTGCGGTACAACCATAATCCAGGAATGGAAAGACTATGTAGGCTGGAGCTGCTTCG |
| *yjjB*-Id-F | GCTAGTGCCGGGCTTTCCGTT |
| *yjjB*-Id-R | CTTCCCGGACGCGCCAGCT |
| *yjfO*-Ex-F | AATTAGATCTATGGTTAGCAGGAAACGTAATAGCG |
| *yjfO*-Ex-R | AATTTCTAGATTATTTACGGTACAAAATGGCTTGT |
| *yjfO*-Ko-F | ATGGTTAGCAGGAAACGTAATAGCGTTATTTACCGGTTTGCCAGTTTATTCTATATGAATATCCTCCTTAG |
| *yjfO*-Ko-R | TTATTTACGGTACAAAATGGCTTGTGAATACCACTGTCCTGTCACGATGGTATGTAGGCTGGAGCTGCTTCG |
| *yjfO*-Id-F | CTGGATGAAGTAAAGCCGTGATAAA |
| *yjfO*-Id-R | TGCGGATCCCCTGAAATATTGCTAA |
| *yeaX*-Ex-F | AATTAGATCTATGTCAGACTATCAAATGTTTGAA |
| *yeaX*-Ex-R | AATTTCTAGA CTACAAATCCAACACCAGGCGT |
| *yeaX*-Ko-F | ATGTCAGACTATCAAATGTTTGAAGTACAGGTGAGCCAGGTTGAACCCCTCTATATGAATATCCTCCTTAG |
| *yeaX*-Ko-R | CTACAAATCCAACACCAGGCGTTTACCCTTCGCACGCGAACAACAGATCATATGTAGGCTGGAGCTGCTTCG |
| *yeaX*-Id-F | TGCAAAACTACGATATTTACTTCAC |
| *yeaX*-Id-R | GCTTAACCTGATGGACATGCCG |
| *yhfK*-Ex-F | AATTAGATCTTTGTTTCCCCCGATGTGG |
| *yhfK*-Ex-R | AATTTCTAGATTACGCCTTCGAATCCCGCA |
| *yhfK*-Ko-F | TTGTTTCCCCCGATGTGGCGCAGACTGATTTATCACCCCGATATCAACTACTATATGAATATCCTCCTTAG |
| *yhfK*-Ko-R | TTACGCCTTCGAATCCCGCAACTTGCGACTCAGCCAAATCCCGTGATGCGTATGTAGGCTGGAGCTGCTTCG |
| *yhfK*-Id-F | TCTGCAAGTCACTTCAGAG |
| *yhfK*-Id-R | GATGTATTTAGCGATATTCG |
| *nuoJ*-Ex-F | AATTAGATCTATGGAGTTCGCTTTTTATATCTGTG |
| *nuoJ*-Ex-R | AATTTCTAGATAATGCGTGCTCCTCCGTTTTTCTT |
| *nuoJ*-Ko-F | ATGGAGTTCGCTTTTTATATCTGTGGCCTGATAGCCATACTTGCGACCTTCTATATGAATATCCTCCTTAG |
| *nuoJ*-Ko-R | TCATGCGTGCTCCTCCGTTTTTCTTTTCGCGCTGTCGTCTTTACGATTGCTATGTAGGCTGGAGCTGCTTCG |
| *nuoJ*-Id-F | TCGCTGGTACCCGGAATTTTT |
| *nuoJ*-Id-R | TTCATCCGCGCATCTCACTTAC |
| *bass*-Ex-F | AATTAGATCTATGCATTTTCTGCGCCGA |
| *bass*-Ex-R | AATTTCTAGATTATATCTGGTTTGCCACGTACTGA |
| *bass*-Ko-F | ATGCATTTTCTGCGCCGACCAATATCGCTGCGCCAACGGCTGATATTGACCTATATGAATATCCTCCTTAG |
| *bass-Ko-R* | TTATATCTGGTTTGCCACGTACTGATCTTTCTTCAGCCGTACCCAGGCCCTATGTAGGCTGGAGCTGCTTCG |
| *bass*-Id-F | CTGATTGTTGGCAATCTGA |
| *bass*-Id-R | GGAAATTCTCGTCGAGC |
| *fis*-Ex-F | AATTAGATCTATGTTCGAACAACGCGTAAATTCTG |
| *fis*-Ex-R | AATTTCTAGATTAGTTCATGCCGTATTTTTTCAAT |
| *fis*-Ko-F | ATGTTCGAACAACGCGTAAATTCTGACGTACTGACCGTTTCTACCGTTAACTATATGAATATCCTCCTTAG |
| *fis*-Ko-R | TTAGTTCATGCCGTATTTTTTCAATTTTTTACGCAGCGTACCACGGTTGATATGTAGGCTGGAGCTGCTTCG |
| *fis*-Id-F | CCTGGATCTTTCGGGAAATC |
| *fis*-Id-R | CACTCTGCAATCACTTCAAACA |
| *sufS*-Ex-F | AATTAGATCTATGATTTTTTCCGTCGACAAAGTGC |
| *sufS*-Ex-R | AATTTCTAGATTATCCCAGCAAACGGTGAATACG |
| *sufS*-Ko-F | ATGATTTTTTCCGTCGACAAAGTGCGGGCCGACTTTCCGGTGCTTTCGCGCTATATGAATATCCTCCTTAG |
| *sufS*-Ko-R | TTATCCCAGCAAACGGTGAATACGTTGCAGGCCGGTCACCAGACGATCCATATGTAGGCTGGAGCTGCTTC |
| *sufS*-Id-F | CATCAAAACGGATGGTCAG |
| *sufS*-Id-R | ATCCTGCGGCGTCATCTG |
| *ymfG*-Ex-F | AATTAGATCTATGCTTCAAATGTTAACTCTT |
| *ymfG*-Ex-R | AATTTCTAGATTACGCGGTCTGGCTGCC |
| *ymfG*-Ko-F | ATGCTTCAAATGTTAACTCTTGAAGAATGGGCTGCTGAAAAATACAGAAGCTATATGAATATCCTCCTTAG |
| *ymfG*-Ko-R | TTACGCGGTCTGGCTGCCATTACTCAAAATCCGTTGCAAAAGTAATGAGTTATGTAGGCTGGAGCTGCTTCG |
| *ymfG*-Id-F | CGACCTTCTGTGGATACCC |
| *ymfG*-Id-R | TTGAGTCTTATATCACCACTTTT |
| *tehB*-Ex-F | AATTAGATCT ATGATCATTCGTGACGAAAACT |
| *tehB*-Ex-R | AATTTCTAGATAATTTTTTACGTGCCAGCATCG |
| *tehB*-Ko-F | ATGATCATTCGTGACGAAAACTATTTTACTGATAAATATGAATTAACCCGCTATATGAATATCCTCCTTAG |
| *tehB*-Ko-R | TCATTTTTTACGTGCCAGCATCGTGGCGAAACGCAGTTTAATACGATTACTATGTAGGCTGGAGCTGCTTCG |
| *tehB*-Id-F | TTTCGGTTATGGACTGCTGC |
| *tehB*-Id-R | AATAGTTTCCTGAGATGAT |
| *cysB*-Ex-F | AATTAGATCTATGAAATTACAACAACTTCGC |
| *cysB*-Ex-R | AATTTCTAGATTATTTTTCCGGCAGTTTT |
| *cysB*-Ko-F | ATGAAATTACAACAACTTCGCTATATTGTTGAGGTGGTCAATCATAACCTCTATATGAATATCCTCCTTAG |
| *cysB-Ko-R* | TTATTTTTCCGGCAGTTTTATATCTTTAAACATGACCTCAATTTCTTCATTATGTAGGCTGGAGCTGCTTCG |
| *cysB*-Id-F | ACAGCAATATGTCTCTTCGGAA |
| *cysB*-Id-R | CAGCAAGACGTTGAACGATTGGTGT |
| *rfaH*-Ex-F | AATTAGATCTATGCAATCCTGGTATTTACTGTACT |
| *rfaH*-Ex-R | AATTTCTAGATTAGAGTTTGCGGAACTCGGTATTC |
| *rfaH*-Ko-F | ATGCAATCCTGGTATTTACTGTACTGCAAGCGCGGGCAACTTCAACGTGCCTATATGAATATCCTCCTTAG |
| *rfaH*-Ko-R | TTAGAGTTTGCGGAACTCGGTATTCTTCACACTGTGCTTAATCTCTTTATTATGTAGGCTGGAGCTGCTTCG |
| *rfaH*-Id-F | GCACGCAAAGTGCGGTC |
| *rfaH*-Id-R | GATGCAGGCGTGCGTGG |
| *rfaP*-Ex-F | AATTGGTACCATGGTTGAATTAAAAGAGCCG |
| *rfaP*-Ex-R | AATTTCTAGATTATAATCCTTTGCGTTGTGTTC |
| *rfaP*-Ko-F | ATGGTTGAATTAAAAGAGCCGCTTGCCACACTTTGGCGTGGTAAAGATGCCTATATGAATATCCTCCTTAG |
| *rfaP*-Ko-R | TTATAATCCTTTGCGTTGTGTTCGCTCTTTAATACGCTCGGCCTTAACGCTATGTAGGCTGGAGCTGCTTCG |
| *rfaP*-Id-F | TATTACTGCATCCCGCTTATC |
| *rfaP*-Id-R | ATTTTTTAATATTTGTTTATTGATA |
| *acrB*-Ex-F | AATTAGATCTATGCCTAATTTCTTTATCG |
| *acrB*-Ex-R | AATTTCTAGATAAATGATGATCGACAGTATGG |
| *acrB*-Ko-F | ATGCCTAATTTCTTTATCGATCGCCCGATTTTTGCGTGGGTGATCGCCATCTATATGAATATCCTCCTTAG |
| *acrB*-Ko-R | TCAATGATGATCGACAGTATGGCTGTGCTCGATATCTTCATTCTTGCGGCTATGTAGGCTGGAGCTGCTTCG |
| *acrB*-Id-F | TTTTAGTCCCGCAACAGGG |
| *acrB*-Id-R | CGCCAGATGAAATTGGTGAC |
| *pgpA*-Ex-F | AATTAGATCTATGACCATTTTGCCACGC |
| *pgpA*-Ex-R | AATTTCTAGACTACGACAGAATACCCAG |
| *pgpA*-Ko-F | ATGACCATTTTGCCACGCCATAAAGATGTCGCGAAAAGTCGCCTGAAGATCTATATGAATATCCTCCTTAG |
| *pgpA*-Ko-R | CTACGACAGAATACCCAGCGGCCAGTGATGACCGATAAAATACAGGATGCTATGTAGGCTGGAGCTGCTTCG |
| *pgpA*-Id-F | CCGATCTCGGGCATATCGT |
| *pgpA*-Id-R | CGATGAAAATGACGCGCAG |
| *acrA*-Ex-F | AATTAGATCTATGAACAAAAACAGAGGGTTTACG |
| *acrA*-Ex-R | AATTTCTAGATTAAGACTTGGACTGTTCAGGCT |
| *acrA*-Ko-F | ATGAACAAAAACAGAGGGTTTACGCCTCTGGCGGTCGTTCTGATGCTCTCCTATATGAATATCCTCCTTAG |
| *acrA*-Ko-R | TTAAGACTTGGACTGTTCAGGCTGAGCACCGCTTGCGGCTTGCTGGTTATTATGTAGGCTGGAGCTGCTTCG |
| *acrA*-Id-F | TTTAAAATGCCAGTAGATTGC |
| *acrA*-Id-R | GTCAGGGTGATCTGCACGGT |
| *torR*-Ex-F | AATTAGATCTATGCCACATCACATTGTTATTG |
| *torR*-Ex-R | AATTTCTAGATAAGCACACATCAGCGGCTAAG |
| *torR*-Ko-F | ATGCCACATCACATTGTTATTGTTGAAGATGAGCCGGTTACCCAGGCGCGCTATATGAATATCCTCCTTAG |
| *torR-Ko-R* | TCAGCACACATCAGCGGCTAAGAAATAACCTTCACCATGTTGCGTCACCATATGTAGGCTGGAGCTGCTTCG |
| *torR*-Id-F | CACCGGTTGCATACTGTGGCAACTG |
| *torR*-Id-R | TGCGGCAGAGGCGGCAATGGGGG |
| *yceA*-Ex-F | AATTGGTACCATGCCAGTGTTACACAACCGC |
| *yceA*-Ex-R | AATTTCTAGATTATTCTGTTGGATCAGGAATGC |
| *yceA*-Ko-F | ATGCCAGTGTTACACAACCGCATTTCCAACGACGCGCTAAAAGCCAAAATCTATATGAATATCCTCCTTAG |
| *yceA*-Ko-R | TTATTCTGTTGGATCAGGAATGCACAGTGTTGTATTCAGACGTCCACGAGTATGTAGGCTGGAGCTGCTTCG |
| *yceA*-Id-F | GTACGCCAATACCCAACCAGGT |
| *yceA*-Id-R | ATTCACCTCCACCAGCGTGAAG |
| *flgE*-Ex-F | AATTAGATCTATGGCCTTTTCTCAAGCGGTT |
| *flgE*-Ex-R | AATTTCTAGATTAGCGTAAGTTAACCAGCGTG |
| *flgE*-Ko-F | ATGGCCTTTTCTCAAGCGGTTAGCGGATTAAACGCTGCCGCCACCAACCTCTATATGAATATCCTCCTTAG |
| *flgE*-Ko-R | TTAGCGTAAGTTAACCAGCGTGTTGAGGATCTGGTCCTGGGTTTTGATGGTATGTAGGCTGGAGCTGCTTCG |
| *flgE*-Id-F | GATAAAAATGGCGCGGTTGTGCG |
| *flgE*-Id-R | TCAGCGGTCTGCACGGCCAG |
| *ybiX*-Ex-F | AATTGGTACCATGATGTACCACATTCCCGG |
| *ybiX*-Ex-R | AATTTCTAGATAAGATCTCCGACCATTCC |
| *ybiX*-Ko-F | ATGATGTACCACATTCCCGGCGTGTTATCGCCACAGGACGTCGCTCGTTTCTATATGAATATCCTCCTTAG |
| *ybiX*-Ko-R | TCAGATCTCCGACCATTCCCGCAGCAGATTATGATAAAGATTAAGCAGCGTATGTAGGCTGGAGCTGCTTCG |
| *ybiX*-Id-F | CTCTGTTGGCGCGGG |
| *ybiX*-Id-R | CCTGTATAAGCTGGTTTTTGTAAAT |
| *yfbP*-Ex-F | AATTGGTACCATGATGAAACTTATTCCTCGCAGT |
| *yfbP*-Ex-R | AATTTCTAGATTAAGTGAACTGTGTACGACTATCG |
| *yfbP*-Ko-F | ATGATGAAACTTATTCCTCGCAGTAGTGATATTTCACCTGGCATTGATGGCTATATGAATATCCTCCTTAG |
| *yfbP*-Ko-R | TTAAGTGAACTGTGTACGACTATCGCCAAAATACTCTAAACATAACTGCCTATGTAGGCTGGAGCTGCTTCG |
| *yfbP*-Id-F | TGAAAATACGTGAGCGTAACAATGT |
| *yfbP*-Id-R | GGTTGTCGGTTCGGCAATCGG |
| *rcsC*-Ex-F | AATTGGTACCTTGAAATACCTTGCTTCTTTT |
| *rcsC*-Ex-R | AATTTCTAGACTACGAATCCCGCGATTTCCT |
| *rcsC*-Ko-F | TTGAAATACCTTGCTTCTTTTCGTACAACCCTGAAAGCCTCGCGCTACAT CTATATGAATATCCTCCTTAG |
| *rcsC*-Ko-R | CTACGAATCCCGCGATTTCCTGACCCTCTCGGCATATAACGTCAGCGTCTTATGTAGGCTGGAGCTGCTTCG |
| *rcsC*-Id-F | AATAGTAAGCGTTGGGACAATGACC |
| *rcsC*-Id-R | AAGAAATCTGCGATGATGAAGCTGG |
| *mpaA*-Ex-F | AATTGGTACC ATGACCGTAACCCGCCC |
| *mpaA*-Ex-R | AATTTCTAGATAACGACGGGCGAATTG |
| *mpaA*-Ko-F | ATGACCGTAACCCGCCCACGCGCCGAACGCGGCGCATTTCCGCCCGGAACCTATATGAATATCCTCCTTAG |
| *mpaA*-Ko-R | TCACGACGGGCGAATTGCATCTTTAGGATGCCAGCGCAGCAAGTTTGCCATATGTAGGCTGGAGCTGCTTCG |
| *mpaA*-Id-F | TTGCTGCCAATAATCTGGGATAATT |
| *mpaA*-Id-R | TTTTATTCATCCGTTGCCGATTTGT |
| *ybaJ*-Ex-F | AATTGGTACCATGGATGAATACTCACCCAAAAGAC |
| *ybaJ*-Ex-R | AATTTCTAGACTAACAAGATAAACTCGCAGGATTC |
| *ybaJ*-Ko-F | ATGGATGAATACTCACCCAAAAGACATGATATCGCACAGCTTAAGTTTCTCTATATGAATATCCTCCTTAG |
| *ybaJ*-Ko-R | CTAACAAGATAAACTCGCAGGATTCTCTTTCGTCGCATTGACAAAACAACATGTAGGCTGGAGCTGCTTCG |
| *ybaJ*-Id-F | TATTACGTCATATTCAGAGCAATCC |
| *ybaJ*-Id-R | AAAAGTATAAAATTCTTAATAAACA |
| *hha*-Ex-F | AATTGGTACCATGTCCGAAAAACCTTTAACG |
| *hha*-Ex-R | AATTTCTAGATTAGCGAATAAATTTCCATACTGAG |
| *hha*-Ko-F | ATGTCCGAAAAACCTTTAACGAAAACCGATTATTTAATGCGTTTACGTCG CTATATGAATATCCTCCTTAG |
| *hha*-Ko-R | TTAGCGAATAAATTTCCATACTGAGGAAGGGATCTTGTCGTACAGTTTATTATGTAGGCTGGAGCTGCTTCG |
| *hha*-Id-F | GTAAACGACCCAACCTCGGCGATCA |
| *hha*-Id-R | GCCAGGGAATGATTGTATCGGTGAA |
| *phoP*-Ex-F | AATTAGATCT ATGCGCGTACTGGTTGTTGAAGAC |
| *phoP*-Ex-R | AATTTCTAGATTAGCGCAATTCGAACAGATAGC |
| *phoP*-Ko-F | ATGCGCGTACTGGTTGTTGAAGACAATGCGTTGTTACGTCACCACCTTAACTATATGAATATCCTCCTTAG |
| *phoP*-Ko-R | TCAGCGCAATTCGAACAGATAGCCCTGGCCGCGAACGGTGGTAATCACTTTATGTAGGCTGGAGCTGCTTCG |
| *phoP*-Id-F | GACGCCGAAGGCATGAAGCAGTTTA |
| *phoP*-Id-R | CACGTTGCGCCCATAAAAGCTGCCC |
| *phoQ-*Ex-F | AATTAGATCT ATGAAAAAATTACTGCGTC |
| *phoQ-*Ex-R | AATTTCTAGATTATTCATCTTTCGGCGC |
| *phoQ-*Ko-F | ATGAAAAAATTACTGCGTCTTTTTTTCCCGCTCTCGCTGCGGGTACGTTTCTATATGAATATCCTCCTTAG |
| *phoQ*-Ko-R | TTATTCATCTTTCGGCGCAGAATGCTGGCGACCAAAAATCACCTCCATCCTATGTAGGCTGGAGCTGCTTCG |
| *phoQ-*Id-F | ACAGGTCATTTCGCTCCCCCCGTTT |
| *phoQ-*Id-R | ACGGGCCGTGGCTGACCTGCCATTT |
| *tolC*-Ex-F | AATTAGATCTATGAAGAAATTGCTCCCCATTCTT |
| *tolC*-Ex-R | AATTTCTAGATTAGTTACGGAAAGGGTTATGACCG |
| *tolC* -Ko-F | ATGAAGAAATTGCTCCCCATTCTTATCGGCCTGAGCCTTTCTGGGTTCAGCTATATGAATATCCTCCTTAG |
| *tolC* -Ko-R | TCAGTTACGGAAAGGGTTATGACCGTTACTGGTGGTAGTGCGTGCGGATGTATGTAGGCTGGAGCTGCTTCG |
| *tolC* -Id-F | ATCTAATGAAAAAAAGCCGC |
| *tolC* -Id-R | GTCAGCATTTTGATAGAGAGAC |
| *pmrK*-Ex-F | AATTGCTAGCATGAAATCGGTACGTTACCTTAT |
| *pmrK*-Ex-R | AATTTCTAGATAATTTGGGACGATACTGAATC |
| *pmrK* -Ko-F | ATGAAATCGGTACGTTACCTTATCGGCCTCTTCGCGTTTATTGCCTGCTACTATATGAATATCCTCCTTAG |
| *pmrK* -Ko-R | TCATTTGGGACGATACTGAATCAGCACCAGACGCTCCTGACGATCGATGGTATGTAGGCTGGAGCTGCTTCG |
| *pmrK* -Id-F | ACCTGGGATGAAGTGATTGGTCGGG |
| *pmrK*-Id-R | CAATAATGAACGCCACCCCACACCA |
|  | **Primers used in EMSA** |
| *folA*-5’-FP | attcagatggtggaagagatggcaatggtt |
| *folA*-1-FP | gatgaaccggaaacgaaaccctcatcctaa |
| *folA*-2-FP | TAAAGAGTGACGTAAATCACACTTTACAGC |
| *folA*-3-FP | TAACTGTTTGTTTTTGTTTCATTGTAATGC |
| *folA*-4-FP | GGCGAGTCCAGGGAGAGAGCGTGGACTCGC |
| *folA*-3’-RP-B | Biotin-GCGTTTAAACCAGGCGAGATCGGCAGGCAG |
| *folA*-1-RP-B | Biotin-TTAGGATGAGGGTTTCGTTTCCGGTTCATC |
| *folA*-2-RP-B | Biotin-GCTGTAAAGTGTGATTTACGTCACTCTTTA |
| *folA*-3-RP-B | Biotin-GCATTACAATGAAACAAAAACAAACAGTTA |
| *folA*-4-RP-B | Biotin-GCGAGTCCACGCTCTCTCCCTGGACTCGCC |
| *folA*-RP-B | Biotin-TGAGATTTCCCGATAAAAAAAATTGTCGCC |
| *folA*-3’-RP-B | Biotin-GCGTTTAAACCAGGCGAGATCGGCAGGCAG |
|  | **Primers used in *lacZ*-fusion construction** |
| P*_folA_*-231/26-F | AAAAGGATCCGGAACCGAAGAAGGTAAA |
| P*_folA_*-181/26-F | AAAAGGATCCGAAACCCTCATCCTAATA |
| P*_folA_*-131/26 -F | AAAAGGATCCTGTTTGTTTTTGTTTCAT |
| P*_folA_*-81/26-F | gatccACTCGCCAGCAGAATATAAAATTTTCCTCAACATCATCCTCGCACCAGTCGACGACGGTTTACGCTTTACGTATAGTGGCGACAATTTTTTTTATCGGGAAATCTCAa |
| P*_folA_*-31/26-F | gatccGACGACGGTTTACGCTTTACGTATAGTGGCGACAATTTTTTTTATCGGGAAATCTCAa |
| P*_folA_*--R | ATATAAGCTTTGAGATTTCCCGATAAAA |
| P*_folA_*-81/26-R | agcttTGAGATTTCCCGATAAAAAAAATTGTCGCCACTATACGTAAAGCGTAAACCGTCGTCGACTGGTGCGAGGATGATGTTGAGGAAAATTTTATATTCTGCTGGCGAGTg |
| P*_folA_*-31/26-R | agcttTGAGATTTCCCGATAAAAAAAATTGTCGCCACTATACGTAAAGCGTAAACCGTCGTCg |
| S2-BamhI-F | aaaaGGATCCataaagagtgacgtaaat |
| dm-BamhI-F | aaaaGGATCCcactttacagctaactgttt |
| HindIII-R | atatAAGCTTTGAGATTTCCCGATAAAA |
| pZT102-F | AGTTGGCAGCATCACCCGA |
|  | **Primers used in Gene replacement** |
| PfolA-rp-F | aaacataccggcaacatggcggatgaaccggaaacgaaaccctcatcctaaATATGAATATCCTCCTTAG |
| PfolA-rp-dm-R | ggactcgccgcattacaatgaaacaaaaacaaacagttagctgtaaagtgTGTAGGCTGGAGCTGCTTCG |
| PfolA-rp-R | ggactcgccgcattacaatgaaacaaaaacaaacagttagctgtaaagtgtgaTTtacgtcactcTTtaTGTAGGCTGGAGCTGCTTCG |
| PfolA-id-F | CAGATGGTGGAAGAGATGGCA |
| PfolA-id-R | TAATCACGGGTTTATTTAAGG |
|  | **Primers used in Foot Prting assay** |
| FP-F | AAACATACCGGCAACATGGCG |
| FP-R-Fam | GTAAAGCGTAAACCGTCGTCG |
|  | **Primers used in Quantitative real-time PCR Assays** |
| gapA-F | TCCCAGAACATCATCCCGTCCTC |
| gapA-R | AACGCCATACCAGTCAGTTTGCC |
| phoQ-F | AACAGATTGCTCTCGCCACGTAAC |
| phoQ-R | ACTGGTGCTTTCGCTTGCCTAC |
| phoP-F | GGGGAGCGAAATGACCTGTGAAG |
| phoP-R | AGTGCCGGTGCTGATGATTATGTG |
| tolC-F | GACCGTGCGTTCCTCCTTCAAC |
| tolC-R | ACCGAGTAGCCCGCTTCCATC |
| folA-F | TGACGCATATCGACGCAGAAGTG |
| folA-R | CCGCCGCTCCAGAATCTCAAAG |
